# Transcriptome Study in Sicilian Patients with Huntington’s Disease

**DOI:** 10.3390/diagnostics15040409

**Published:** 2025-02-07

**Authors:** Michele Salemi, Vincenzo Di Stefano, Francesca A. Schillaci, Giovanna Marchese, Maria Grazia Salluzzo, Angela Cordella, Ilenia De Leo, Concetta Simona Perrotta, Giuseppe Nibali, Giuseppe Lanza, Raffaele Ferri

**Affiliations:** 1 Oasi Research Institute-IRCCS, 94018 Troina, EN, Italy; fran7.sch@gmail.com (F.A.S.); msalluzzo@oasi.en.it (M.G.S.); or giuseppe.lanza1@unict.it (G.L.); rferri@oasi.en.it (R.F.); 2 Department of Biomedicine, Neuroscience and Advanced Diagnostics (Bi.N.D.), University of Palermo, 90127 Palermo, PA, Italy; vincenzo19689@gmail.com; 3 Genomix4Life S.r.l., 84081 Baronissi, SA, Italy; giovanna.marchese@genomix4life.com (G.M.); angela.cordella@genomix4life.com (A.C.); ileniadeleo87@gmail.com (I.D.L.); 4 Genome Research Center for Health-CRGS, 84081 Baronissi, SA, Italy; 5 Vittorio Emanuele Gela Hospital, Asp 2 of Caltanissetta, 93012 Gela, CL, Italy; kettyperrotta@yahoo.it; 6 U.O.S.D. Neurology and Stroke Unit, P.O. Umberto I, 96100 Siracusa, SR, Italy; giuseppenibali@gmail.com; 7 Department of Biomedical and Biotechnological Sciences, University of Catania, 95125 Catania, CT, Italy

**Keywords:** mRNAs, RNA sequencing, Huntington’s disease, transcriptome analysis

## Abstract

**Background/Objectives**: Huntington’s disease (HD) is an autosomal dominant neurodegenerative disorder caused by the expansion of the CAG nucleotide repeat in the first exon of the huntingtin (*HTT*) gene. The disease typically manifests between the second and third decades of life and progresses gradually. The pathogenesis of HD involves the dysregulation of gene expression, influenced by various molecular processes ranging from transcription to protein stability. **Methods**: To investigate potential variations in gene expression associated with HD, a transcriptome study was conducted using peripheral blood mononuclear cell samples from 15 HD patients and 15 controls, all of Sicilian origin. **Results**: The analysis identified 7179 statistically significant differentially expressed genes between the two groups. Gene Set Enrichment Analysis (GSEA) and Gene Ontology (GO) terms were applied to identify the pathways affected by these differentially expressed mRNAs. The GSEA results highlighted significant associations between HD and GO pathways related to ribosomal functions and structure. These pathways were predominantly characterized by negative expression, with a substantial number of genes showing dysregulation. This suggests that the molecular processes leading to protein translation via ribosomes may be impaired in HD. Furthermore, dysregulation was observed in genes and non-coding RNAs involved in regulatory roles across various transcriptional processes. **Conclusions**: These findings support the hypothesis that the entire process, from transcription to translation, is disrupted in HD patients carrying the CAG repeat expansion in the first exon of the *HTT* gene.

## 1. Introduction

Huntington’s disease (HD) is an autosomal dominant neurodegenerative disorder caused by the expansion of the CAG nucleotide repeat in the first exon of the huntingtin (*HTT*) gene [1]. The disease typically manifests in the second or third decade of life and progresses slowly, presenting with neurological symptoms such as chorea, psychiatric disturbances, cognitive decline, dysphagia, and characteristic gait disorders with frequent falls [2]. HD remains a rare and orphan disease, with no effective treatments currently available. Management primarily involves symptomatic therapies, physiotherapy, and complication prevention [2,3].

The pathogenesis of HD is linked to the dysregulation of gene expression, influenced by molecular processes ranging from transcription to protein stability. Although the genetic cause of HD is well defined, additional genetic factors may modify the progression of this Mendelian monogenic disorder [4]. Among these modifiers, mismatch repair genes are notably implicated in CAG repeat instability, impacting the age of disease onset. Interestingly, FANCD2 and FANCI-associated nuclease 1 (*FAN1*), previously unrelated to repeat instability, has shown the strongest HD-modifying effects.

In this context, oligonucleotide-based therapeutics, such as short interfering RNA (siRNA), offer a potential strategy to reduce HTT mRNA and protein levels in vivo. However, the non-selective reduction of wild-type HTT poses risks, potentially compromising gene function and therapeutic efficacy [5,6]. Transcriptome-wide mRNA sequencing may help identify allele-selective small RNAs capable of specifically targeting mutant *HTT*. The central and peripheral nervous systems require tightly regulated protein synthesis for proper neuronal function, a requirement particularly critical in neurodegenerative and triplet-repeat expansion disorders [7,8,9].

In HD, CAG repeat expansions may disrupt the entire protein synthesis machinery, affecting processes in the nucleus and cytoplasm where ribosomes translate mRNA into proteins. Such disruptions may alter cellular activity and overall protein production, triggering nucleolar stress and activating cell death pathways [9]. Notably, the down-regulation of rRNA transcription, a hallmark of nucleolar dysfunction, has been implicated in nucleolar stress and cell death in polyQ disorders like spinocerebellar ataxias and HD [10].

This highlights the need for ongoing research to optimize genetically encoded repeat-targeted small RNAs for allele-selective HD therapies. Changes in the architecture of the protein synthesis machinery may underlie translational disruptions in HD, necessitating detailed investigation to inform disease management and therapy development [11].

To explore these dynamics, we conducted a transcriptome study on a cohort of Sicilian HD subjects, genetically diagnosed with CAG triplet expansion in the *HTT* gene’s first exon. The data were compared with controls (CTRs) to identify the genetic pathways most affected in HD.

## 2. Materials and Methods

### 2.1. RNA Extraction

RNA was analyzed from the peripheral blood mononuclear cells (PBMCs) of 15 HD subjects (9 males, 6 females; mean age 52.43 ± 15.98 years) and 15 controls (11 males, 4 females; mean age 46.33 ± 20.37 years). PBMCs were separated using Ficoll-Paque (Ficoll-Plaque PLUS-GE Healthcare Life Sciences, Piscataway, NJ, USA), and total RNA was isolated with TRIzol reagent (Invitrogen Life Technologies, Carlsbad, CA, USA) following the manufacturer’s protocol. RNA was stored at −80 °C until analysis. Concentration and purity were measured using the NanoDropOne (Thermo Fisher Scientific, Waltham, MA, USA), and integrity was assessed using the TapeStation 4200 (Agilent Technologies, Santa Clara, CA, USA) [12].

### 2.2. Ethics Approval and Consent

Informed consent was obtained from all participants or their relatives, as required. This study adhered to the Declaration of Helsinki and was approved by the Ethics Committee of the Oasi Research Institute-IRCCS of Troina, Italy, on 4 May 2021 (2021/05/04/CE-IRCCSOASI/43).

### 2.3. RNA Sequencing and Data Analysis

RNA sequencing and data analysis were conducted by Genomix4Life S.r.l. (Baronissi, Italy). Libraries were prepared from 800 ng of purified RNA using the Illumina Stranded mRNA Prep Kit (Illumina, San Diego, CA, USA) and quantified with the TapeStation 4200 (Agilent Technologies, Santa Clara, CA, USA) and Qubit 4 fluorometer (Thermo Fisher Scientific, Waltham, MA, USA). These libraries were pooled in equimolar amounts and sequenced on the Illumina NovaSeq6000 platform (2 × 101 paired-end format).

Raw sequence files (.fastq format) underwent quality control via FastQC. Cutadapt (v.2.8) [13] was used to remove low-quality reads, short sequences (≤25 bp), and adaptor sequences. The processed fastq files were aligned to the reference genome using the STAR bioinformatics tool (version 2.7.3a) [14], with default settings for paired-end reads. The human genome assembly from GenCode (hg38 release 37 (GRCh38.p13)) served as the reference. Gene expression levels for each sample were calculated using the featureCounts algorithm [15]. Data normalization was performed in R using negative binomial generalized linear models through the Bioconductor DESeq2 package, considering all genes expressed in ≥25% of samples. This method accounts for both the average expression level and gene dispersion, ensuring the accurate estimation of expression differences between conditions. The Bioconductor DESeq2 package employs the Benjamini–Hochberg (BH) method to calculate adjusted *p*-values (padj) with default settings, controlling the false discovery rate (FDR), which represents the expected proportion of false positives among significant results [16]. Genes with a fold change ≥ 2.50 or ≤−2.50 (|FC| ≥ 2.50) and adjusted *p*-values ≤ 0.001 (padj ≤ 0.001), as recommended by the literature [16,17,18], were considered differentially expressed. Heatmaps and volcano plots of these differentially expressed genes were generated using the ComplexHeatmap version 2.14.0 [19] and ggplot2 version 3.5.1 [20] packages in R, respectively.

### 2.4. Functional and Pathways Analysis of Differentially Expressed Genes

To investigate the pathways influenced by differentially expressed mRNAs (DEmRNAs) and identify significant changes in HD patients, we performed Gene Set Enrichment Analysis (GSEA) to determine whether predefined gene sets exhibit statistically significant and coordinated differences between two biological conditions. The differentially expressed genes were analyzed by calculating the enrichment scores of specific gene sets across various functional categories to detect notable and consistent differences between the two states. For this analysis, we focused on the Gene Ontology (GO) C5 module from the Molecular Signature Database (MSigDB), querying three primary ontologies: molecular functions (MFs), biological processes (BPs), and cellular components (CCs).

A functional analysis of the significant genes was carried out using GSEA (v.4.1.0) [21], a computational tool that evaluates RNA-Seq data at the gene set level. GSEA determines whether predefined gene sets exhibit significant, consistent differences between two conditions. The gene sets for this analysis were obtained from MSigDB and included genes categorized by their HGNC gene symbols, ensuring the use of standardized and globally recognized gene nomenclature. This approach provides a comprehensive understanding of the biological processes, pathways, and functional categories involved in the conditions under study. The results offer insights into crucial biological mechanisms and highlight the most relevant gene sets contributing to the observed gene expression differences.

Cytoscape version 3.9.1 [22] was employed to visualize the selected GO terms, enabling the construction of complex networks. This software facilitated the creation and visualization of networks, where nodes represent genes or gene products, and edges signify the biological relationships between them. GO terms related to categories such as biological processes, molecular functions, and cellular components were mapped onto the networks to emphasize key interactions and pathways. Cytoscape’s advanced layout and visualization tools were utilized to optimize the network representation, making it easier to interpret the connections and the biological significance of the involved genes.

## 3. Results

### 3.1. Whole Transcriptome of Huntington’s Patients

**Figure 1:**
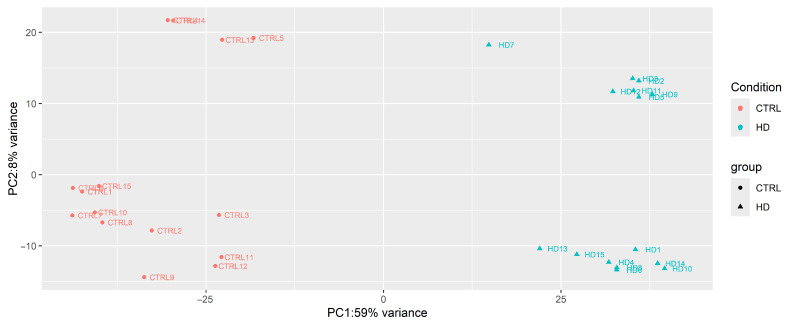
The principal component analysis of transcripts found in HD patients and controls showing the clustering of the two groups by using the first principal component (PC1) and the second (PC2).

Whole-transcriptome RNA sequencing was conducted using next-generation sequencing in patients and controls with the aim of elucidating potential differences in gene expression between the two groups. Principal component analysis (PCA) (Figure 1) was performed, revealing significant variance between the groups and a clear separation between sample conditions.

**Figure 2:**
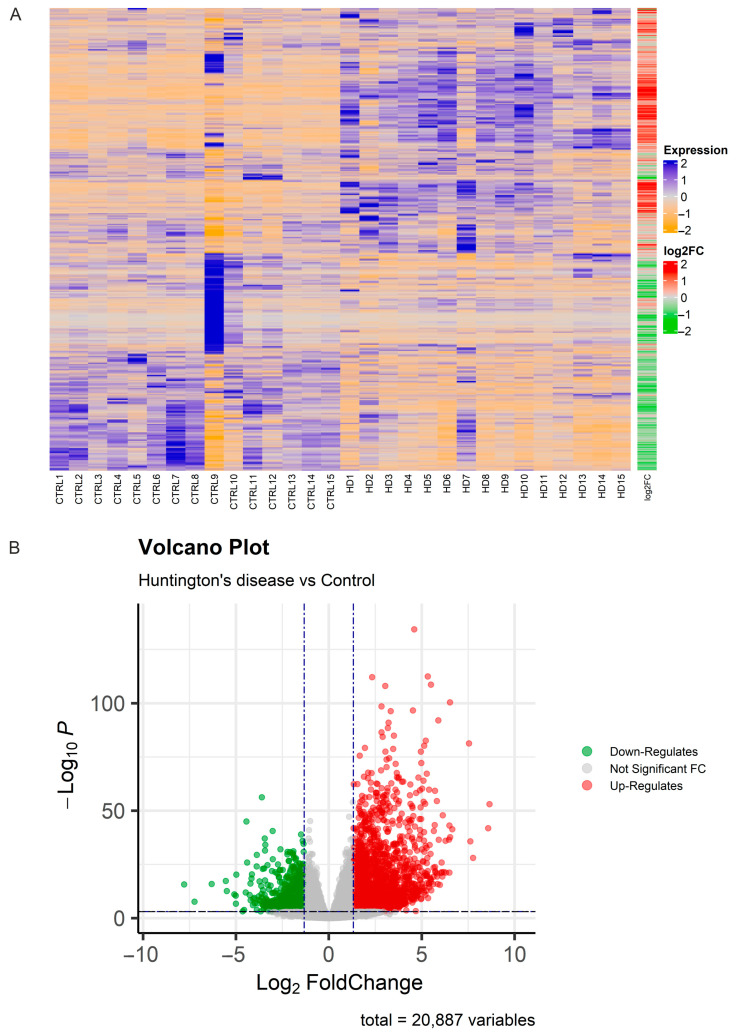
Differentially expressed genes (DEGs). (**A**) Heatmap illustrating significant DEGs in patients with Huntington’s disease (HD) compared to CTRL individuals. Genes with increased expression levels are depicted in blue, while those with decreased expression levels are shown in orange. log2 (foldChange) bar indicates up-regulated genes in red and down-regulated genes in green; (**B**) volcano plot representing significant DEGs based on fold changes and *p*-values. Down-regulated genes are depicted in green, while up-regulated genes are shown in red. Up/down-regulated in HD.

Gene expression levels were quantified using featureCounts with common gene annotations, identifying a total of 20,887 expressed genes across all samples. Hierarchical clustering analysis was employed to investigate overall gene expression differences, demonstrating consistent trends in gene expression changes between the two groups, with statistically significant differences observed (Figure 2A). Differential expression analysis identified 7179 genes as statistically significant (padj ≤ 0.001) between the two groups (Huntington vs. control). Among these, 1985 genes were significantly up-regulated (padj ≤ 0.001 and FC ≥ 2.5), while 694 genes were significantly down-regulated (padj ≤ 0.001 and FC ≤ −2.5) (Supplementary Table S1). Volcano plots depicted distinct differences in gene regulation between the treated and control groups (Figure 2B). Raw data and normalized gene counts are available on ArrayExpress under accession number E-MTAB-14696.

### 3.2. The Gene Set Enrichment Analysis (GSEA)

Our dataset consisted of 2679 native features used for the analysis. We applied gene set size filters (minimum = 15, maximum = 500), which led to the removal of 14,643 out of 16,008 gene sets, following the default GSEA settings. This left 1365 gene sets for further analysis. The analysis report is divided into two sections under “Enrichment in Phenotype”: the first section highlights the results for gene sets with a positive enrichment score (indicating enrichment at the top of the ranked list), while the second section presents the results for gene sets with a negative enrichment score (indicating enrichment at the bottom of the ranked list) (Supplementary Tables S2 and S3).

**Figure 3:**
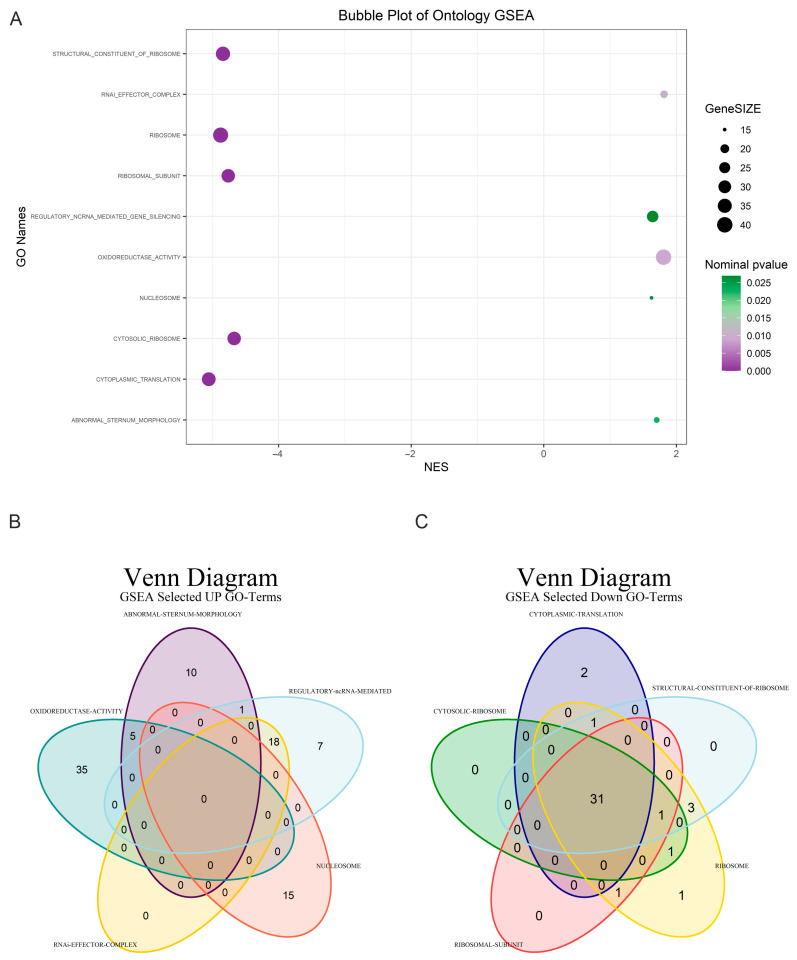
Gene Set Enrichment Analysis (GSEA) plots. (**A**) Bubble plot depicting selected Gene Ontology (GO) terms enriched in GSEA. Each GO term is represented by three distinct numerical parameters: Normalized Enrichment Score (NES), nominal *p*-value, and gene set size. In light green, more significant nominal *p*-values are indicated. (**B**) Venn diagram illustrating specific and common up-regulated transcripts among five selected GO terms. (**C**) Venn diagram demonstrating specific and common down-regulated transcripts among five selected GO terms.

Of the 1365 gene sets analyzed, 559 were up-regulated in the “na_pos” phenotype (positive enrichment score). None of these gene sets were significant at an FDR < 25%, with one gene set showing significant enrichment at a nominal *p*-value < 1% and six gene sets significantly enriched at a nominal *p*-value < 5%. In the “na_neg” phenotype (negative enrichment score), 806 gene sets were up-regulated. Notably, 436 gene sets showed significant enrichment at an FDR < 25%, 237 were significantly enriched at a nominal *p*-value < 1%, and 335 were significantly enriched at a nominal *p*-value < 5%. Among the GO terms (Figure 3A), we focused on the top five GO terms that were up-regulated in the “na_pos” phenotype.

In detail,

- GOMF_Oxidoreductase_Activity (Normalized Enriched Score (NES 1.8));
- GOCC_RNAI_Effector_Complex (NES 1.8);
- HP_Abnormal_Sternum_Morphology (NES 1.7);
- GOCC_Nucleosome (NES 1.6);
- GOBP_Regulatory_ncRNA_Mediated_Gene_Silencing (NES 1.6).

The pathway GOMF_Oxidoreductase_Activity (NES 1.8) refers to enzymes responsible for catalyzing oxidation–reduction reactions, crucial for cellular processes such as metabolism, energy production, and detoxification. GOCC_RNAI_Effector_Complex (NES 1.8) involves the RNA-induced silencing complex, a key player in RNA interference (RNAi), which regulates gene expression by targeting and degrading specific mRNA molecules, contributing to gene silencing and viral defense. HP_Abnormal_Sternum_Morphology (NES 1.7) describes abnormalities in the sternum’s structure, often linked to developmental or genetic disorders, potentially manifesting as skeletal deformities. GOCC_Nucleosome (NES 1.6) refers to the nucleosome, a core unit of chromatin structure, essential for DNA packaging and regulating gene expression through its interaction with histone proteins. Lastly, GOBP_Regulatory_ncRNA_Mediated_Gene_Silencing (NES 1.6) highlights the role of non-coding RNAs (ncRNAs) in silencing genes by targeting mRNA for degradation or inhibiting its translation, a process crucial for development, differentiation, and disease prevention. Conversely, we also studied the top five GO terms in the “na_neg” phenotype, including GOBP_Cytoplasmic_Translation (NES −5.0); GOCC_Ribosome (NES −4.8); GOMF_Structural_Constituent_of_Ribosome (NES −4.8); GOCC_Ribosomal_Subunit (NES −4.7); and GOCC_Cytosolic_Ribosome (NES −4.6).

The pathway GOBP_Cytoplasmic_Translation (NES −5.0) involves the process of protein synthesis within the cytoplasm, where ribosomes translate messenger RNA (mRNA) into proteins. This process is crucial for cell function and growth, as proteins are essential for most cellular activities. A negative enrichment score suggests that cytoplasmic translation is down-regulated, which can impact overall protein production and cellular activity. GOCC_Ribosome (NES −4.8) refers to the ribosome, the molecular machine responsible for translating mRNA into proteins. Ribosomes consist of two subunits (large and small) and are found either floating in the cytoplasm or attached to the endoplasmic reticulum. A down-regulation in ribosome activity can reduce protein synthesis, affecting numerous biological processes, especially in cells that rely heavily on rapid protein production, such as proliferating cells. GOMF_Structural_Constituent_of_Ribosome (NES −4.8) points to the molecular function of ribosomal proteins that form the core structure of ribosomes.

GOCC_Ribosomal_Subunit (NES −4.7) pertains to the individual large or small subunits of the ribosome, which work together during translation. A negative enrichment score here suggests a reduction in the availability or function of these ribosomal subunits, further compromising the cell’s ability to produce proteins. GOCC_Cytosolic_Ribosome (NES −4.6) refers specifically to ribosomes located in the cytosol, which are responsible for translating proteins that function within the cytoplasm. It is interesting to note that the GO terms associated with the “na_pos” phenotype do not share any common genes, while those related to the “na_neg” phenotype display a significant overlap in genes, as illustrated in the Venn diagrams shown in Figure 3B.

**Figure 4:**
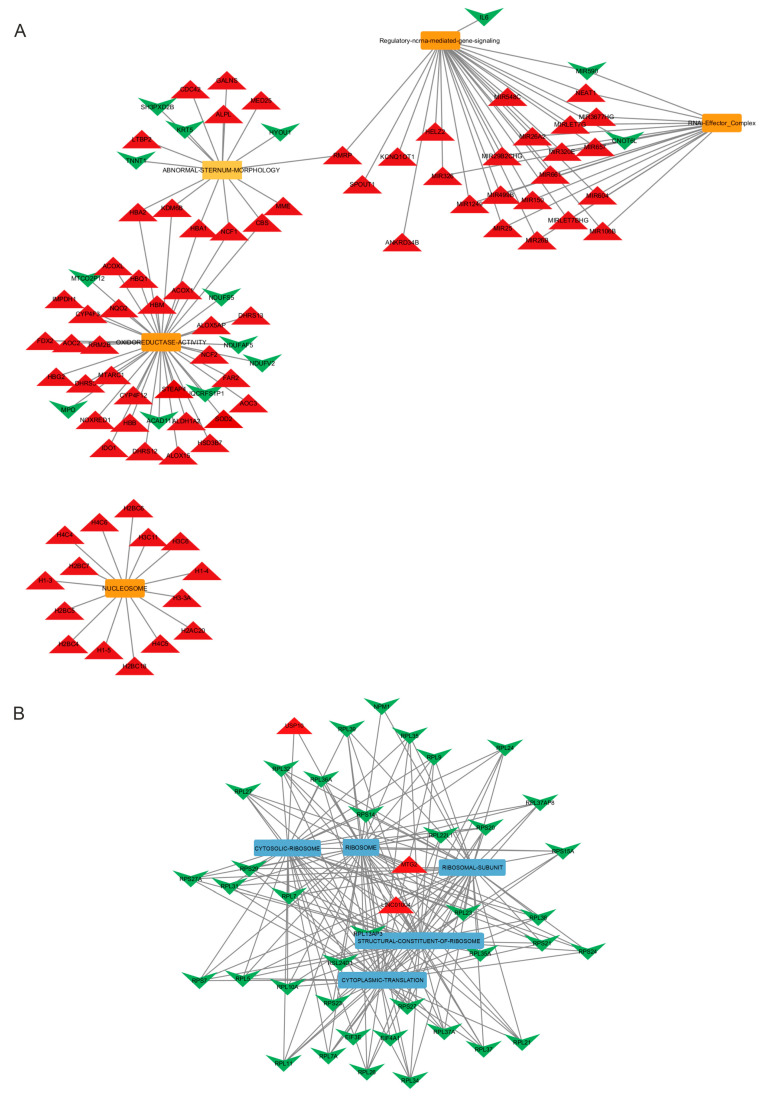
The network constructed using Cytoscape illustrates the relationship between dysregulated genes and GSEA pathways. Nodes in the network represent the Gene Ontology (GO) terms: (**A**) yellow nodes indicate positive Normalized Enrichment Scores (NESs), (**B**) while blue nodes indicate negative NESs. The green color denotes down-regulated genes, while the red color indicates up-regulated genes.

Cytoscape analysis was conducted to further elucidate the connections between the selected GO terms and the genes exhibiting differential expression across various categories of biological processes, molecular functions, or cellular components. As depicted in Figure 4, each GO term is represented as a node, with genes linked to them as edges. 

By integrating gene expression data with GO term annotations and representing them as a network, we can highlight functional relationships between genes and the specific biological processes or pathways in which they participate. This deeper understanding aids in elucidating the underlying mechanisms governing biological processes.

## 4. Discussion

### 4.1. GO Terms in the “na_neg” Phenotype

Both the central and peripheral nervous systems are extremely susceptible to the spatial and temporal control of protein synthesis, and a malfunction in the ribosomal complex, ribosomal subunits (80S, 40S minor subunit, and a 60S major subunit), and/or in the conformation of the polyribosome are involved in the development and progression of several neurodegenerative diseases [7,8,9]. Of note, the top five negative phenotypes highlighted by GO are all related to the ribosome structure and function.

It is worth noting that patients with HD showed a reduced expression of two similar pathways, i.e., “RIBOSOME” and “CYTOSOLIC_RIBOSOME”, along with “CYTOPLASMIC_TRANSLATION”, compared to age-matched healthy individuals. In this context, a very recent preclinical study demonstrated that polyglutamine [polyQ]-mediated ribotoxicity disrupts proteostasis and stress responses in HD [23]. In particular, both the translation and aggregation of the wild-type *HTT* gene and mutant *HTT* gene (mHTT) were regulated by a stress-responsive upstream open reading frame, and polyQ expansions caused the abortive translation termination and release of truncated, aggregation-prone mHTT fragments. Notably, mHTT depleted translation elongation factor eIF5A in the brains of symptomatic HD mice and cultured HD cells and caused pervasive ribosome pausing and collisions, eventually leading to disrupted homeostatic controls and recovery failure from acute stress [23]. Therefore, drugs that inhibit translation initiation may reduce premature termination and arrest this escalating cascade of ribotoxic stress and dysfunction in HD.

In another recent study, ribosome profiling and mass spectrometry revealed widespread mitochondrial translation defects in a striatal cell model of HD, with many mitochondrial transcripts having comparable or higher ribosome occupancy but with decreased mitochondrial protein products in HD [24]. Of note, ribosomal transcription is regulated by PGC-1alpha, a versatile inducer of mitochondrial biogenesis and responsive to the changing energy demands of the cell, and impaired in HD. Translationally, this novel molecular link between ribosomal and mitochondrial biogenesis helps to explain sarcopenia and cachexia in diseases of neurodegenerative origin, such as HD [25]. Although adequate translation in humans is still lacking, CAG repeat instability in both the peripheral and central nervous systems of transgenic HD monkeys has recently been reported. The correlation analysis of CAG repeat expansion and the gene expression profile of ribosomal protein lateral stalk subunit P1 and ribosomal protein L13a, among other genes, showed a strong correlation with CAG repeat instability [26].

Nucleolar stress and dysfunction have also been linked to the pathogenesis of HD, since alterations in nucleolar activity and integrity seem to contribute to the deregulation of ribosomal DNA transcription in HD pathogenesis [27]. This is not surprising given that the nucleolus, a dynamic nuclear biomolecular condensate and the site of ribosomal RNA transcription, is implicated in cellular stress response and protein quality control in several neurodegenerative diseases, including HD [28]. Indeed, the down-regulation of rRNA transcription is a common cause of nucleolar function disruption, which subsequently triggers nucleolar stress, and has been associated with the pathogenesis of poly-Q-related neurological disorders such as HD and spinocerebellar ataxias [29]. Interestingly, the expression of expanded CAG transcripts is able to directly trigger nucleolar stress in HD [10]: In an R6/2 HD transgenic mouse model, expanded mHTT transcripts were found to physically interact with nucleolin, a nucleolar protein that plays a crucial role in ribosome biogenesis. Then, mHTT transcripts prevented nucleolin from binding onto the Upstream Control Element (UCE) of the ribosomal RNA promoter. This resulted in UCE hypermethylation, which abolished the binding of the transcription factor Upstream Binding Factor to UCE and subsequently led to the down-regulation of pre-45s rRNA transcription. Furthermore, the p53/mitochondria-dependent nucleolar stress cell death pathway was activated in polyQ diseases, such as HD [10].

Finally, very recently, Martin-Solana et al. [30] conducted an electron tomography study in striatal tissue in HD zQ175 knock-in mice, focusing on the polyribosome. Specifically, they found that ribosome stalling was associated with altered polyribosome architecture, which was found to be denser and more compact, with the overexpression of eukaryotic translation initiation factor 5A2 (EIF5A2) and eukaryotic translation initiation factor 5A (EIF5A1). They hypothesized, as a result, that these alterations may be vital for the development of HD-related phenotypes and for the progression of the disease itself.

Lastly, it is worth mentioning that even in 1977, Prashad and Rosenberg conducted a study of skin fibroblasts in a cohort of HD subjects and healthy controls with the aim of assessing the presence or absence of ribosomal protein alterations in order to identify some possible diagnostic markers. Using the one-dimensional sodium dodecyl sulfate polyacrylamide gel electrophoresis technique, they found no difference in the ribosomal proteins of skin fibroblasts in the study cohort, consequently assuming that there was no binding dysfunction between ribosomal RNAs (rRNAs) and the ribosomal proteins themselves, as this would be easily demonstrated and manifested [31].

Overall, these findings indicate that changes in the architecture of the protein synthesis machinery may underlie translational alterations associated with HD, thus opening new avenues for understanding the progression and management of the disease [11]. In particular, increased translation due to the dysregulation of protein synthesis is involved in mHTT-induced striatal neuron dysfunction and may be recognized as a novel pathogenic mechanism in HD [32]. In this scenario, proteomic characterization indicates that translation specifically affects sets of proteins, i.e., the up-regulation of ribosomal and oxidative phosphorylation proteins and the down-regulation of proteins related to neuronal structure and function. Accordingly, treatment with the translation inhibitor 4EGI-1 prevented R6/1 mouse motor deficits, although cortico-striatal long-term depression was not markedly changed in behaving animals. At the molecular level, the injection of 4EGI-1 normalized protein synthesis and ribosomal content in R6/1 mouse striatum [32]. To summarize, mHTT stalls ribosomes and represses protein synthesis in HD; thus, mHTT impedes ribosomal translocation during translation elongation, a mechanistic defect that can be exploited for HD therapeutics. This is fully in line with the reduced expression of the ribosomal pathways and cytoplasmic translation we observed in the present study, although further validation in humans is needed.

### 4.2. GO Terms in the “na_pos” Phenotype

The positive regulation of GOBP_Regulatory_ncRNA_Mediated_Gene_Silencing in HD compared to controls appears to be more complex to explain, although evidence pointed out the transcriptional dysregulation of both coding and non-coding genes in cellular models of HD. Previous work has highlighted REST (RE1 (repressor element 1)-silencing transcription factor) as one such transcription factor. REST is a master regulator of neuronal genes, repressing their expression. Many of its direct target genes are known or suspected to have a role in HD pathogenesis, including the Brain-Derived Neurotrophic Factor. Recent evidence has also shown that REST regulates the transcription of regulatory miRNAs (microRNAs), many of which are known to regulate neuronal gene expression and are dysregulated in HD. Thus, the repression of miRNAs constitutes a second indirect mechanism by which REST can alter the neuronal transcriptome in HD [33]. Since these original observations were made, several thousand direct target genes of REST have been identified, including numerous non-coding RNAs, including both miRNAs and long non-coding RNAs, several of which are dysregulated in HD.

More recently, evidence is emerging that hints at epigenetic abnormalities in HD brains [34]. This, in turn, promotes the notion that targeting the epigenetic machinery may be a useful strategy for the treatment of some aspects of HD. REST also recruits a host of histone- and chromatin-modifying activities that can regulate the local epigenetic signature in REST target genes. Similarly, human accelerated region 1 (HAR1), a rapidly evolving cis-antisense locus that is specifically transcribed in the nervous system, is repressed by REST in HD, and consistent with other REST target genes, HAR1 levels are significantly lower in the striatum of HD patients compared with unaffected individuals [35]. Collectively, these observations present REST as a hub that coordinates transcriptional, post-transcriptional, and epigenetic programs, many of which are disrupted in HD [34].

Based on these observations, HD represents an ideal candidate for gene silencing with oligonucleotide therapeutics, i.e., antisense oligonucleotides (ASOs) and small interfering RNAs (siRNAs). Using an ultra-sensitive branched fluorescence in situ hybridization (FISH) method, it has been shown that approximately 50% of wild-type HTT mRNA localizes to the nucleus and that its nuclear localization is observed only in neuronal cells [36]. In mouse brain sections, HTT mRNA was detected predominantly in neurons, with a wide range of HTT foci observed per cell. Both siRNAs and ASOs efficiently eliminated cytoplasmic HTT mRNA and HTT protein, but only ASOs induced a partial but significant reduction in nuclear HTT mRNA. As such, like other mRNAs, HTT mRNA subcellular localization might play a role in important neuronal regulatory mechanisms [36].

We showed positive expressions of GOCC_NUCLEOSOME. Eukaryotic DNA is organized into chromatin, a structure that controls the essential functions of the genome. The basic unit of chromatin is called the nucleosome. The nucleosome consists of ~147 base pairs (bp) of DNA wrapped around a complex of histone octamers, each histone octamer containing two copies each of histones H2A, H2B, H3, and H4 [37,38]. Human genomic DNA is wrapped in ~30 million nucleosomes, connected by DNA linkers ranging in length from ~20 to 70 bp [39,40] and compacted into higher-order dynamic structures [38,41,42]; this organization allows for accessibility to the genome to control transcription, recombination, DNA repair, replication, kinetochore and centromere formation, and so forth [43,44]. There are 14 contact points between histones and DNA [37]. These multiple interactions make the nucleosome one of the most stable protein–DNA complexes under physiological conditions; because of this, it is well suited for its packaging function. However, the nucleosome is not a simple static unit, rather it has dynamic properties that are tightly regulated by various protein complexes [44].

Based on these observations, it is clear how nucleosomes are the key structures in the mechanisms enabling gene transcription and how the dysregulation of genes that code for nucleosome composition is of pivotal importance in pathogenetic mechanisms, such as in this case of HD. Accordingly, in our study, 15 genes of the nucleosome pathway are shown to be dysregulated.

RNA interference (RNAI) is a complex, highly conserved mechanism of gene expression regulation, also present in mammalian cells, mediated by small double-stranded interfering RNAs (siRNAs), which are the effector molecules of gene silencing [45,46,47]. Specifically, these siRNAs are oligonucleotides that are identified by RNAI effector molecules, leading to gene silencing (sequence-specific cleavage of messenger RNA (mRNA), translational repression, transcript degradation) [45,46]. RNAIs have so far been evaluated only for therapeutic use, precisely to reduce or silence gene expression, due to knowledge of the nucleotide sequence gel target gene [45]. As early as 2004, Xia et al. [48] conducted a study on spinocerebellar ataxia type 1 (SCA1), a polyglutamine expansion disease, such as HD disease and others. They evaluated, in particular, RNAIs for therapeutic use in a mouse model, and from this, they noted both cellular and behavioral improvement in a guinea pig, making use of the repression of mutant allele expression.

Chen et al. [49] set out to conduct a study with the aim of reducing the expression of Htt through the development of a combination of RNAIs and gene transfer carried out by non-viral Sleeping Beauty (SB) transposons. Next, they quantified the transcribed and translated HTT gene via a real-time polymerase chain reaction (RT-PCR) and Western blot analysis, respectively. Eventually, they found that siRNA constructs significantly reduced the mRNA and protein levels of Htt compared with controls. Others, such as McBride et al. [50] and Boudreau et al. [51], evaluated, separately, the functionality of RNAIs to achieve a protein reduction in Htt in transgenic, i.e., genetically modified, mice, finding effective protein silencing with an overall improvement for the organism. Tabrizi et al. [52] published a review on possible therapeutic developments for HD. They found that administering RNAIs or ASOs, all the way to the brain, is difficult; small molecules that can cross the blood–brain barrier and are easily administered should be identified.

In our study, we identified this cellular component as positively regulated, hypothesizing that its action is extrinsic with gene silencing that could play a positive role in individuals with HD or even make the disease more progressive and aggressive. From Figure 4, one can see the relationship between dysregulated genes and GSEA pathways, such as the over-expression of the Nuclear Enriched Abundant Transcript 1 (*NEAT1*) gene also identified by Johnson in 2012 [53].

Murthy et al. [54] observed that the *HTT* gene plays roles during the various stages of an organism’s development, and the resulting dysfunctions are not well understood. Therefore, they set out to observe and describe the effects that a progressive reduction in *HTT* gene expression could develop in an *HTT* mouse model, also clarifying the gene networks that are dysregulated during organ development. In conclusion, they found that organisms presenting inactivating mutations of the *HTT* gene show abnormalities in body size, skin, ear skeletal formation, and hematopoiesis defects, which led them to think that HTT plays a fundamental role in supporting normal embryonic and fetal development. HP_Abnormal Sternum Morphology is a phenotype that has been little studied and detected in HD subjects and requires further research in the medical–scientific field to better understand the positive regulation of this phenotype.

GOMF_Oxidoreductase_Activity refers to the catalysis of an oxidation–reduction (redox) reaction, a reversible chemical reaction in which one substrate acts as a hydrogen or electron donor and is oxidized, while the other acts as a hydrogen or electron acceptor and is reduced. Fox et al. [55] expanded their previous study in which they had recorded that the N-terminal 171 fragment of mHTT was prone to thiol oxidation, and they hypothesized that this within mHTT may contribute to cellular accumulation and toxicity in individuals with HD. From their evidence and others’ in the scientific literature, they assumed that there may be a specific thiol-disulfide oxidoreductase present that leads to a reduction in mHTT in cells, thus providing protection to the mouse model of HD. Through genetic screening, they detected two proteins, thioredoxin 1 and thioredoxin-related transmembrane protein 3 (TRX1 and TRX3), capable of lowering mHTT levels in both cultured cells and induced striatal neuronal atrophy. Using this in the present study, this finding seems to support their evidence for a possible role of dysregulated protein-thiol homeostasis in the pathogenesis of HD [56].

Several studies in the literature have focused on transcriptional profiling between individuals with HD and healthy controls, conducted on model systems such as mouse and cell lines, as well as on biological samples like peripheral blood and postmortem human brain tissues. Changes in gene expression were observed across all sample types. HD is recognized as a systemic disease because the HTT protein is expressed in all cells and tissues [57,58,59].

Labadorf et al. [58] conducted a transcriptomic study of the postmortem human prefrontal cortex, specifically focusing on Brodmann area 9 (BA9), which, along with the striatum, is implicated in HD pathogenesis. They found a predominance of overexpressed genes, with approximately two-thirds coding for proteins, while the rest comprised long non-coding RNAs (lincRNAs), pseudogenes, and antisense transcripts. These genes were associated with immune response, neuroinflammation, and developmental processes, suggesting their potential for developing HD therapies.

Our findings show a similarity in the presence of differentially expressed miRNAs compared to the aforementioned data. Eshraghi et al. [60] demonstrated that mutant HTT (mHtt) promotes ribosome stalling, suppressing protein synthesis in mouse striatal HD neuronal cells. Specifically, the depletion of mHtt positively influences protein synthesis, whereas its presence hinders ribosomal elongation and translocation during protein synthesis. This suggests that ribosome stalling may contribute to the progressive and widespread development of HD-related symptoms. Developing drugs targeting ribosome stalling mechanisms could be a promising therapeutic strategy for HD. This study partially correlates with our data on ribosomal mRNAs.

Andrade-Navarro et al. [61] conducted a transcriptomic study to monitor disease progression and treatment response. They identified significant differences in gene expression between HD patients and controls, highlighting the dysregulation of immune system and inflammatory response functions. They concluded that changes in gene expression in peripheral blood might reflect disease-associated immune pathology. Some of the differentially expressed genes in our study also correlate with inflammatory and immune mechanisms (see Figure 4). This supports the potential validity of peripheral blood studies in HD and other neurodegenerative diseases.

Hensman Moss et al. [62] demonstrated that transcriptome studies on peripheral blood mirror the findings from transcriptome studies on the brains of HD subjects. This correlation has also been observed in Alzheimer’s disease, indicating that these neurodegenerative diseases are systemic and not confined to the brain. This evidence suggests that differences in gene expression in peripheral blood could also reflect the changes occurring in the brains of the same patients.

### 4.3. Translational Considerations

From a translational perspective, the findings reported here could offer potential clinical applications or therapeutic insights. For instance, developing models to assess nucleolar stress in HD and evaluating therapeutic strategies to mitigate its effects would be valuable [28]. Histone deacetylase inhibitors, which have shown promise in improving the HD phenotype by promoting DNA transcription, are one such potential strategy [28]. Similarly, drugs targeting the polyglutamine expansion of huntingtin-mediated ribosome stalling mechanisms may help prevent or slow the progression of HD [60].

A further exploration of other nucleolus-associated proteins affected by neurological diseases, including HD, is promising. We emphasize the importance of studying specific gene expression dysregulation in HD pathogenesis to understand the underlying mechanisms of mutant huntingtin toxicity, which could reveal new therapeutic opportunities [63]. Additionally, the observed changes in non-coding RNA expression in HD warrant further investigation. Understanding their potential mechanisms and molecular functions, particularly their roles in transcriptional regulation, could provide deeper insights into HD pathogenesis, identify possible biomarkers, and uncover new therapeutic targets [64,65].

However, the role of epigenetic alterations in HD pathogenesis remains to be fully elucidated. Future studies should address whether these alterations are critical markers of HD disease progression and could serve as objective outcome measures [66].

## 5. Conclusions

The enrichment analysis revealed the decisive role of ribosomal dysfunction in HD, thus suggesting the impairment of the chain leading to protein translation (via ribosomes) from mRNA. This implies that a large part of the catalytic functions of the cells of individuals with HD appears to be impaired, a concept that had already been demonstrated in the work of Eshraghi et al. [60]. Similarly, we showed the dysregulation of genes and ncRNAs that play a regulatory role in the various transcription processes involved in HD. Overall, these findings confirm that the entire process from transcription to translation is impaired in HD subjects genetically diagnosed with a CAG triplet expansion of the first exon of the *HTT* gene. Notwithstanding some intrinsic limitations, this might be obtained through the study of the relatively specific genetic signature of HD from peripheral blood mononuclear cells, thus providing an accurate diagnostic frame for the early detection and reliable monitoring of HD patients. Translationally, further enhancing our understanding of the genetic basis of HD will contribute to improved diagnostic accuracy, therapeutic developments, and prognostic insights for affected individuals.

## Data Availability

Raw data and normalized gene counts are available at ArrayExpress under accession number E-MTAB-14696.

## Supplementary Materials

The following supporting information can be downloaded at https://www.mdpi.com/article/10.3390/diagnostics15040409/s1, Table S1: The table shows the genes that differential expression analysis detected between the two groups (Huntington vs. control) 1985 genes significantly up-regulated (padj ≤ 0.001 and FC ≥ 2.5) while 694 genes significantly down-regulated (padj ≤ 0.001 and FC ≤ -2.5); Table S2: The table highlights the analysis ratio of the “Enrichment in Phenotype” section for the results of gene sets with a positive enrichment score; Table S3: The table highlights the analysis ratio of the “Enrichment in Phenotype” section for the results of gene sets with a negative enrichment score.
