# Supplementary material for: Transcriptome Study in Sicilian Patients with Huntington’s Disease
Source: Diagnostics (Basel). 2025 Feb 7;15(4):409. doi: 10.3390/diagnostics15040409 (PMC11854416; doi:10.3390/diagnostics15040409)
Supplement: Supplementary file 1 [file diagnostics-15-00409-s001.zip › Supplementary_Table S1.pdf]

Supplementary Table S1. The table shows the genes that differential expression analysis detected between the two groups (Huntington vs. control) 1985 genes significantly up-regulated (padj ≤ 0.001 and FC ≥ 2.5) while 694 genes significantly down-regulated (padj ≤ 0.001 and FC ≤ -2.5).

Gene ID	log2FoldChange	padj
CXCL2	-7.78374872	2.18E-16
CCL3L1	-7.224786064	2.24E-08
AREG	-6.305509987	1.46E-16
CXCL8	-5.545547909	4.97E-18
G0S2	-5.486389683	2.45E-13
ENSG00000273338	-5.08649353	8.48E-12
ENSG00000261026	-5.00591455	3.45E-11
CXCL3	-4.997063264	1.94E-07
RPS27P17	-4.973312995	5.70E-21
CCL20	-4.63772969	7.18E-04
DNAAF1	-4.5540188	1.60E-04
ID1	-4.468167431	1.28E-12
ENSG00000272256	-4.433654571	1.16E-45
RGS1	-4.397114824	1.40E-26
EREG	-4.260749059	1.73E-11
CD83	-4.212704996	8.04E-17
MT-TV	-4.206165201	5.30E-09
FOSB	-4.162878612	4.13E-19
EGR1	-4.141904501	4.24E-21
ENSG00000242660	-4.084162684	1.00E-06
NR4A3	-4.0638692	1.21E-09
RPL7A	-4.010090592	3.71E-10
RPS15AP17	-3.984057382	1.17E-07
NR4A2	-3.882514522	9.49E-25
IL6	-3.874411883	1.59E-04
RPS27P9	-3.872953748	3.99E-30
EIF3LP2	-3.852930037	1.44E-07
RPL4P3	-3.774610937	8.10E-11
EEF1B2P2	-3.755481296	3.44E-09
ENSG00000241494	-3.724453085	9.10E-18
ENSG00000289350	-3.695190788	8.30E-09
ADRA2B	-3.665106719	6.96E-04
ENSG00000213315	-3.63756827	1.22E-15
CHRM4	-3.618910094	5.71E-12
CD69	-3.604844026	6.20E-57
ENSG00000286035	-3.600652315	3.20E-10
RPS27AP1	-3.554753592	7.10E-07
JUN	-3.552394265	1.27E-23
BCLAF1P2	-3.551985054	7.40E-07
RPL30-AS1	-3.537470669	1.10E-13
ENSG00000279274	-3.519766926	1.44E-06
ENSG00000218565	-3.513410321	6.78E-05

NT5C1A	-3.512776965	3.51E-08
ENSG00000244245	-3.481860634	7.95E-24
PTX3	-3.480763994	6.22E-09
MT-TF	-3.452771511	1.42E-11
RPS3AP47	-3.449305505	8.64E-38
TNFAIP3	-3.433536068	2.70E-18
DUSP2	-3.432659205	3.56E-32
RPL21P53	-3.414804888	8.40E-35
ATP5F1EP2	-3.40581813	1.21E-06
HNRNPA1P10	-3.395293012	4.30E-07
SCGB3A1	-3.39328847	5.71E-08
RPL7P47	-3.374377435	7.41E-10
RPL24P6	-3.363406083	2.87E-05
RPL29P12	-3.341739659	5.49E-05
ENSG00000228830	-3.332609018	5.31E-08
WDR38	-3.315859618	2.69E-06
RPL23P8	-3.313242648	3.88E-09
RPL15P2	-3.309797019	9.77E-09
HBEGF	-3.298498023	2.20E-25
EEF1A1P35	-3.290239921	3.03E-16
ASB12	-3.262214942	2.88E-07
TKTL2	-3.25747427	3.23E-05
THBS1-IT1	-3.248095709	3.95E-04
ENSG00000226532	-3.236591432	7.82E-07
ENSG00000289447	-3.224145827	1.18E-04
NFKBIA	-3.217845077	3.85E-14
TPT1P6	-3.215259789	8.98E-08
RPL19P16	-3.206424176	1.89E-05
ENSG00000277998	-3.198907469	1.20E-06
ENSG00000254336	-3.177862024	1.46E-06
SMIM43	-3.16348005	1.06E-04
ENSG00000257386	-3.148451827	1.03E-04
RPS4XP3	-3.140775129	6.63E-05
RPL36AP13	-3.135429742	2.27E-07
ENSG00000264577	-3.127416006	7.11E-16
SNAI1	-3.114188838	2.75E-08
RPS27P7	-3.101179936	2.49E-07
RPS27AP11	-3.097243404	4.81E-08
RPS27P6	-3.094463343	2.25E-05
SIK1	-3.093901552	7.36E-04
RPS4XP6	-3.076742768	2.28E-08
ENSG00000275993	-3.061876597	1.32E-22
RPL39P15	-3.061712088	3.86E-08
ENSG00000229567	-3.052630612	1.07E-08
ZNF208	-3.047109812	2.14E-12
ESAM-AS1	-3.042344147	3.86E-04
RPS23P8	-3.019835748	2.90E-41

RPL13AP7	-3.016121774	4.11E-28
NR4A1	-2.990737077	1.92E-12
ENSG00000213875	-2.988215047	7.82E-04
RPL13AP3	-2.967336466	1.47E-04
ENSG00000269951	-2.957490204	1.25E-07
ENSG00000234718	-2.952034812	4.62E-05
SMAD6	-2.951175437	5.06E-09
ENSG00000242477	-2.929841011	4.21E-05
CCIN	-2.920717651	4.14E-05
ENSG00000239719	-2.914752305	3.63E-06
SYDE2	-2.91100506	1.31E-12
PSPC1P1	-2.888886068	8.09E-04
ENSG00000213280	-2.884287453	6.47E-06
RPL17P39	-2.882374279	4.62E-22
ENSG00000272483	-2.873420737	6.49E-04
PLCH1	-2.85527794	1.93E-05
ENSG00000289474	-2.834642972	2.20E-06
ENSG00000240652	-2.831124392	1.48E-07
RPL21P119	-2.829972552	3.57E-08
B3GNT7	-2.827139155	1.89E-13
RPS29P5	-2.824943278	1.09E-05
HSP90AA2P	-2.823844902	1.08E-04
EEF1A1P22	-2.823175153	4.45E-09
ENSG00000240036	-2.80526364	5.09E-10
PDE4D	-2.768559456	1.91E-21
ENSG00000244086	-2.762497966	1.78E-04
RPL13P2	-2.747083333	7.19E-05
THBS1	-2.745058553	1.97E-09
RPL37P2	-2.736527334	9.95E-26
RPL36AP8	-2.727056014	1.52E-04
RPS4XP11	-2.719808307	5.43E-04
RPL31P49	-2.699896288	1.88E-04
ENSG00000289974	-2.690932532	2.43E-04
RPS26P15	-2.69014843	6.06E-04
TAMALIN	-2.689837982	2.58E-12
EEF1A1P9	-2.687103348	1.19E-19
ENSG00000266356	-2.684217453	1.02E-04
PER1	-2.680202018	5.96E-20
RPL34P18	-2.676470801	8.99E-08
SSBP3-AS1	-2.675390976	4.67E-06
NACA3P	-2.668075628	4.45E-07
FOSL1	-2.66802542	2.23E-08
ENSG00000272432	-2.651322689	1.05E-05
ENSG00000290033	-2.649381331	3.75E-11
ENSG00000259483	-2.647969626	2.66E-08
RPL10P1	-2.642542356	1.39E-06
MTCO2P12	-2.639472738	4.63E-23

RPS26P58	-2.636853301	2.97E-08
DAZAP2P1	-2.631834006	2.46E-04
EXOC3L2	-2.631207809	2.03E-04
C15orf48	-2.629598741	6.56E-04
SNORD59A	-2.626077769	8.13E-11
RASGEF1B	-2.623159717	1.43E-17
UBE2FP1	-2.619868308	7.71E-07
ENSG00000185641	-2.61317449	4.38E-23
EEF1A1P13	-2.603135215	9.58E-33
FAM81B	-2.602030836	1.40E-04
SREBF2-AS1	-2.599979768	3.90E-05
ENSG00000287419	-2.588624889	3.78E-06
EEF1B2P6	-2.578291419	2.30E-09
EEF1A1P24	-2.574908696	3.85E-04
RPL37AP8	-2.572835275	6.79E-04
TMSB4XP1	-2.572549721	5.78E-05
DUSP4	-2.565563845	1.10E-14
ZNF334	-2.562016752	9.22E-04
ENSG00000279456	-2.542058215	2.26E-05
OSM	-2.541211283	1.20E-10
RANBP17	-2.541100686	5.40E-04
ENSG00000288826	-2.537906119	5.09E-09
PRECSIT	-2.532136748	7.17E-04
RPL35P2	-2.5205679	1.08E-09
NACA2	-2.519458947	4.60E-04
CDKN1C	-2.511727089	1.49E-04
ENSG00000250321	-2.511553087	6.26E-10
ENSG00000289193	-2.510804313	2.67E-06
ENSG00000214748	-2.509923408	1.59E-11
HYOU1	-2.509216	3.95E-05
FOS	-2.507004639	1.73E-17
RPL23AP2	-2.50647181	1.56E-09
RPL37P23	-2.494396159	4.03E-07
MIR762	-2.492425351	1.13E-04
ENSG00000273450	-2.49084089	8.72E-04
LTF	-2.490704431	1.48E-05
SEC14L2	-2.48569732	2.16E-11
RPL35P1	-2.480866915	4.77E-06
ENSG00000220472	-2.479699216	3.52E-23
ENSG00000259635	-2.466225375	1.12E-04
MTCO1P2	-2.461511728	1.24E-04
LIMCH1	-2.456962282	1.09E-04
ENSG00000244380	-2.443055794	9.02E-04
CCDC68	-2.436201186	9.73E-04
ENSG00000178715	-2.434113013	7.44E-06
IGSF3	-2.431046377	2.98E-04
SNORD19	-2.426131991	1.25E-04

ENSG00000288999	-2.420657585	2.67E-13
DYTN	-2.420369366	3.03E-05
RPL7AP31	-2.41837819	9.29E-05
PSMD10P1	-2.413922881	2.87E-11
RPL17P18	-2.399343325	1.52E-05
AVPI1	-2.384462683	2.65E-14
SERBP1P5	-2.382019734	7.91E-04
RPL5P1	-2.377138424	1.16E-16
LINC01088	-2.37646915	1.33E-04
EEF1A1P11	-2.375954912	4.17E-14
ENSG00000219928	-2.375089736	1.97E-07
MATN1	-2.374707699	4.41E-04
RPS3AP5	-2.371588893	7.03E-06
ENSG00000285531	-2.370294074	1.47E-12
ZNF704	-2.370113644	1.18E-06
STARD4-AS1	-2.3654382	4.74E-07
CD44-AS1	-2.359062743	2.99E-04
NOCT	-2.351518643	6.58E-11
ENSG00000273565	-2.343777038	7.48E-05
ELOCP19	-2.341963023	5.29E-09
RPL26P6	-2.340913323	2.43E-16
ERRFI1	-2.340762036	5.39E-08
RPL7AP20	-2.340046013	3.25E-04
RPL6P19	-2.336583277	4.29E-07
EEF1A1P4	-2.334500937	1.93E-09
ENSG00000236710	-2.331379283	6.46E-05
RPL21P134	-2.328390528	3.24E-09
SKOR1	-2.328232081	1.10E-07
PET100P1	-2.324078073	1.96E-04
RPS24P19	-2.321570129	3.70E-11
OLR1	-2.321020803	4.52E-04
RPS10P3	-2.314601277	5.07E-11
KRT2	-2.306321832	4.33E-08
RPL21P46	-2.304742865	4.42E-07
ENSG00000222043	-2.301946567	6.13E-06
RPS27AP5	-2.298700693	1.31E-19
RPL15P22	-2.291567656	4.86E-08
RPL5P17	-2.283028241	5.16E-04
NAP1L5	-2.266440666	9.63E-11
PALM	-2.265636665	5.26E-05
ENSG00000273149	-2.265143431	4.90E-16
RPS3AP20	-2.261376534	9.16E-05
NUDT11	-2.259922719	9.76E-07
RPL29P26	-2.258724939	2.65E-09
RPL21P19	-2.257271005	6.61E-05
CREM	-2.255303865	1.87E-12
BZW1P2	-2.248398057	1.06E-06

ENSG00000279319	-2.244753232	7.25E-04
RPL24P8	-2.244741638	9.60E-07
RPS29P3	-2.243726599	6.62E-04
RPL32P18	-2.243648731	9.62E-20
RPL13AP25	-2.232297078	1.75E-20
ENSG00000256913	-2.231269239	2.30E-04
KLF11	-2.229686813	2.13E-30
ENSG00000233406	-2.228409264	1.84E-05
RPS18P5	-2.219267263	1.46E-31
RPS20P14	-2.218305136	3.17E-08
ENSG00000289130	-2.212700243	3.76E-13
ENSG00000244327	-2.206065934	7.17E-05
ENSG00000274104	-2.204239203	7.08E-15
ENSG00000243829	-2.200732683	1.02E-08
TNFSF9	-2.20072135	4.15E-07
HERC2P9	-2.199978309	8.24E-05
ENSG00000289384	-2.190111889	2.23E-06
RPS15AP38	-2.189155699	9.01E-06
RPS26P31	-2.184926852	1.81E-04
ZNF295-AS1	-2.173563458	5.47E-08
ENSG00000219023	-2.169927362	3.66E-13
FFAR1	-2.168117595	3.20E-06
GREM2	-2.165949567	2.82E-06
PIK3R1	-2.163656706	2.75E-19
EEF1B2P3	-2.160385811	2.40E-27
CCR6	-2.158864275	4.70E-08
LINC01619	-2.158624966	4.14E-12
RPL36AP37	-2.152875971	6.10E-18
ENSG00000233264	-2.145958936	7.62E-07
RPL39P3	-2.144978292	8.96E-16
RPL10AP6	-2.141478891	2.30E-27
DPPA4	-2.129273024	5.28E-08
RPL31P4	-2.127807285	2.77E-14
RPS15AP1	-2.115739572	1.59E-11
ENSG00000278991	-2.114789408	7.48E-05
PRKAR2B	-2.114208238	8.32E-07
DDIT4	-2.103057724	3.64E-15
NCR3LG1	-2.096884715	1.06E-12
CTSG	-2.092045214	1.46E-04
RPL7P9	-2.090752751	3.76E-22
PEG10	-2.088868289	3.52E-11
CDKN1A	-2.088258781	2.97E-14
SBDSP1	-2.082174016	1.86E-31
SYNM	-2.082108255	1.36E-05
KRT86	-2.080771169	2.39E-04
MAST4-AS1	-2.073425835	4.17E-04
ACOT1	-2.071302933	4.90E-09

RPS24P8	-2.059594956	3.12E-04
RPSAP12	-2.058790784	2.06E-08
ACSBG1	-2.053973978	3.15E-05
RPLP0P9	-2.05124295	3.38E-23
RPL8P4	-2.04690681	1.24E-06
EEF1A1P7	-2.040656826	9.69E-05
ENSG00000272434	-2.039747717	5.26E-10
DLEU2L	-2.038138553	2.99E-05
DACT3	-2.035088469	2.88E-04
RPL17P7	-2.029729005	7.58E-16
ENSG00000257764	-2.027769158	1.98E-06
LINC02915	-2.021393701	4.89E-05
TNNT1	-2.018485684	7.09E-06
RPL22P1	-2.012053962	1.12E-09
ZNF521	-2.011359567	1.77E-05
ENSG00000287124	-2.011255339	6.69E-05
EEF1A1P19	-1.999985368	1.27E-10
RPS27P3	-1.998637208	9.24E-25
NTN4	-1.99462891	1.10E-04
ENSG00000290737	-1.985088151	1.41E-10
ENSG00000277654	-1.984790497	2.22E-05
ENSG00000231747	-1.983752375	1.44E-13
ENSG00000262873	-1.979761333	2.52E-04
SEC14L5	-1.97935579	3.03E-04
RPS7P1	-1.975281636	5.69E-20
DDX47	-1.97093404	9.89E-19
COL5A2	-1.969323509	4.46E-04
RPL32P16	-1.967447975	1.23E-05
ENSG00000241280	-1.965491755	9.26E-04
RPL3P7	-1.963332956	8.85E-10
KRT5	-1.962897967	4.06E-08
ENSG00000287925	-1.961890457	2.14E-04
NDUFV2	-1.960950486	6.08E-10
ENSG00000288843	-1.959798934	1.86E-05
RPL21P80	-1.959288714	5.61E-18
ACTG1P3	-1.958690564	9.18E-08
ENSG00000232811	-1.957334176	2.07E-05
MT-TI	-1.956483921	9.46E-13
RPS3AP21	-1.9545286	1.26E-07
ENSG00000273306	-1.952113944	4.32E-08
RPL23AP74	-1.949885055	9.87E-11
MAFB	-1.939227056	1.18E-16
SBDS	-1.937385583	6.70E-31
ENSG00000233558	-1.932806901	4.54E-04
RPL34	-1.929127267	1.27E-24
DMD	-1.926902077	7.31E-04
ENSG00000237214	-1.924103964	2.43E-09

ENSG00000289320	-1.921967254	2.36E-09
RPL26P19	-1.921071318	1.76E-10
LINC02728	-1.920374294	4.87E-06
NT5C3AP2	-1.917961723	1.39E-08
SPRY1	-1.917441418	5.68E-06
CFAP161	-1.91706259	2.86E-04
AUTS2	-1.916601648	4.49E-19
CLECL1P	-1.912880346	4.75E-09
BTG3	-1.909339949	6.49E-09
KLF9	-1.909023384	3.29E-30
CLEC1B	-1.905534837	2.62E-08
ENSG00000235859	-1.903978102	9.58E-25
RABGGTB	-1.899819476	8.62E-32
RPL21P16	-1.896179211	6.24E-21
ELF3	-1.895530439	2.41E-09
TBC1D22A-DT	-1.893454561	1.60E-10
SPAG4	-1.887966019	6.14E-05
RPL35P5	-1.883576011	4.88E-06
MT-TT	-1.882766307	1.09E-13
ST7-AS1	-1.882721676	3.08E-04
RPL14P1	-1.879644718	6.47E-29
EAF2	-1.87760144	2.14E-14
LGALS2	-1.87561471	2.29E-15
ENSG00000257511	-1.874585895	6.88E-10
RPS3AP6	-1.874386644	8.52E-23
TCEA1P2	-1.871185497	8.40E-04
ENSG00000272777	-1.871083518	6.25E-05
LRRC32	-1.869002792	2.56E-04
RSPH4A	-1.863895311	2.19E-05
ENSG00000270100	-1.861919888	9.18E-07
RPL17P6	-1.860676344	4.49E-14
SNORA66	-1.858923469	2.81E-07
RPL23AP65	-1.856388804	5.35E-04
TNFRSF21	-1.855058803	1.18E-09
AKT3	-1.853583868	4.13E-04
RPL7AP30	-1.84966773	1.27E-06
RPS3AP26	-1.848240236	1.42E-13
RPL23	-1.847248913	9.67E-24
ENSG00000244313	-1.84562209	5.64E-16
RPL7AP62	-1.840454447	1.80E-04
DCTN6-DT	-1.838572332	1.93E-17
ENSG00000273419	-1.831841502	5.93E-06
ZMAT1	-1.830513504	1.17E-13
FAM182A	-1.82551839	4.60E-04
RPL7P1	-1.824798814	7.98E-07
LINC02390	-1.824224193	7.13E-09
RPS7P3	-1.822929693	5.47E-05

TMSB4XP4	-1.818824998	6.83E-24
SNRPGP10	-1.816171858	7.93E-10
MIR6774	-1.815879052	3.88E-04
GCSAML	-1.815732348	2.75E-14
OVCH1-AS1	-1.811399795	1.87E-04
ENSG00000269968	-1.81039329	5.44E-15
ZNF331	-1.803935817	1.68E-09
RPS15P4	-1.802048012	4.55E-28
RPS27AP16	-1.800132486	2.18E-20
SCN3A	-1.796153983	6.62E-05
ENSG00000255847	-1.792924954	9.45E-15
RPS2P5	-1.788841572	5.65E-06
THEM5	-1.782272661	1.78E-04
RPL6P10	-1.779928383	5.15E-10
EGR3	-1.779073703	1.40E-06
IL6ST-DT	-1.778439135	1.66E-04
NQO2-AS1	-1.775034878	4.19E-05
EIF4BP7	-1.769452025	5.73E-06
APBB2	-1.764993081	6.94E-05
RPL23AP42	-1.764947246	1.59E-28
MCAM	-1.76440769	9.81E-04
RPL41P5	-1.763431055	8.08E-05
ENSG00000261114	-1.756910474	2.33E-06
SNORA3B	-1.756353531	5.46E-12
HNRNPA1P7	-1.755207069	2.61E-08
CXCR4	-1.752937136	2.59E-16
SEM1	-1.752005592	1.71E-25
RPL26	-1.741608733	1.71E-16
MBTPS1-DT	-1.740798917	6.15E-08
PRICKLE2	-1.74061357	6.71E-04
SOCS3	-1.739121652	9.29E-06
AKAP5	-1.735696374	1.21E-07
MPO	-1.734624302	6.33E-15
ENSG00000259869	-1.730207542	5.31E-04
ZFP1	-1.728215987	1.65E-06
EPHA2	-1.728178513	1.48E-07
ENSG00000233967	-1.728014599	8.81E-10
RETN	-1.726576333	7.63E-06
ENSG00000180211	-1.723060887	1.54E-04
EMP1	-1.71873805	2.19E-08
RPS24	-1.716880788	7.67E-19
ENSG00000273174	-1.715858251	2.84E-10
ENSG00000289306	-1.711804323	2.65E-06
NDUFAF5	-1.709302559	1.82E-12
UQCRB	-1.707194163	1.97E-17
RPL9	-1.707115785	4.38E-19
ENSG00000266993	-1.705986905	1.18E-06

RPL31	-1.705906108	1.42E-20
ENSG00000261641	-1.704234764	1.67E-05
RPL3P4	-1.703506048	1.46E-18
RAB13	-1.701636729	7.37E-07
RPS13P2	-1.694632349	6.40E-15
YES1	-1.690850514	7.26E-07
ENSG00000234648	-1.689675917	3.10E-05
C2orf88	-1.686837302	4.18E-11
ZBTB10	-1.686649271	8.72E-11
DUSP1	-1.682991904	1.08E-08
RPS18P12	-1.679534479	7.95E-08
ENSG00000278022	-1.678990307	4.84E-04
CNBD2	-1.677717453	9.30E-10
ENSG00000230979	-1.674150153	6.02E-18
LINC02345	-1.668236318	2.78E-04
GCOM1	-1.665556355	5.69E-04
CTLA4	-1.665380478	4.68E-16
ENSG00000261770	-1.660072758	1.71E-07
GAS2L3	-1.659569215	1.31E-05
RPL36A	-1.659123207	1.03E-19
ENSG00000227077	-1.658428414	6.25E-04
FAT4	-1.656252426	5.45E-05
PLXNA4	-1.654616323	1.23E-10
TIPARP-AS1	-1.643594744	1.09E-11
RAP1BL	-1.641360637	3.17E-05
RPL5	-1.640907369	1.71E-18
MMD	-1.64085088	6.83E-14
ATP2B1-AS1	-1.639892308	6.76E-05
RPS3P3	-1.63547228	5.43E-09
EEF1A1P12	-1.633361942	4.67E-06
RPS23	-1.632546135	6.69E-23
RPL39	-1.631511496	9.00E-23
NPM1P27	-1.631505181	1.28E-16
THRB	-1.631344988	6.35E-04
P2RY1	-1.630366222	1.59E-07
RAMP1	-1.630195996	2.59E-09
DIRAS1	-1.629370537	1.44E-04
CCT8P1	-1.628105795	1.79E-06
ENSG00000232176	-1.626896469	8.59E-07
CCDC59	-1.625739993	8.11E-18
NPM1	-1.624950205	6.24E-21
RUNX3	-1.624825782	1.78E-16
KLHL11	-1.622415339	2.31E-09
NR1D2	-1.622136713	3.27E-16
RPS27A	-1.614542445	1.12E-18
MN1	-1.61387778	2.35E-04
ENSG00000284946	-1.613226569	4.47E-05

DCTN6	-1.612196077	4.63E-11
PFDN4	-1.61193084	5.91E-12
ENSG00000251600	-1.608304667	6.44E-06
RPS27P8	-1.608190266	5.96E-08
RPL15P3	-1.603218673	9.11E-22
HUS1B	-1.602985294	7.20E-05
ZNF628	-1.602650392	8.97E-15
RPL37	-1.600360515	1.08E-21
CAVIN3	-1.596146982	7.60E-04
ZNF256	-1.595532743	3.19E-13
EPB41L4A-AS1	-1.590598897	3.50E-26
NIPA1	-1.590572335	2.16E-05
ENSG00000289587	-1.589687995	1.46E-04
ENSG00000274943	-1.584967362	9.73E-05
LGR4	-1.583720053	1.17E-04
NFKBIZ	-1.580916582	1.33E-07
SNORD100	-1.579932407	5.51E-07
HNRNPLP2	-1.578713828	9.61E-04
ENSG00000225721	-1.577931519	1.96E-06
LACTB2-AS1	-1.575965424	2.02E-06
ERMN	-1.575519684	1.32E-05
RNASE4	-1.57425814	8.33E-04
MTND5P11	-1.573424297	6.18E-06
KLRB1	-1.573029312	8.25E-15
RPL7	-1.569800763	1.58E-18
EGR2	-1.569785078	5.10E-05
ENC1	-1.569437694	3.01E-07
ZNF639	-1.566365869	7.14E-17
KLF10	-1.564210648	7.71E-13
UTF1	-1.563227335	1.11E-04
ASTL	-1.563226503	2.33E-04
SPX	-1.561554919	6.57E-06
JUNB	-1.560540075	3.21E-13
PPP2R5CP	-1.559331721	4.66E-05
SNHG8	-1.557526281	3.37E-23
CABP5	-1.557033239	1.19E-06
RPL7AP50	-1.556371281	3.67E-06
ENSG00000286136	-1.554785468	3.15E-04
MAPK6	-1.552831036	7.35E-15
TTC3P1	-1.552658506	9.61E-04
RPL35A	-1.551774459	1.06E-21
ENSG00000247134	-1.550573433	9.41E-11
FAM110B	-1.549945284	1.18E-06
IRS1	-1.541680125	6.40E-09
ZFAND2A	-1.541595884	5.08E-26
RPS14	-1.540224685	1.42E-22
VEPH1	-1.540112786	2.69E-04

MIR1282	-1.53520678	6.95E-04
LRRC39	-1.532086263	2.53E-05
KCNH8	-1.524363944	7.60E-06
RPS2P46	-1.522842628	1.51E-20
NPIPB4	-1.522711043	1.98E-06
SH3PXD2B	-1.522679217	3.85E-05
LRRFIP1P1	-1.52171071	3.10E-04
LHFPL6	-1.520612265	6.65E-04
STARD4	-1.516203022	5.82E-18
DNAJC19P5	-1.510439426	5.54E-04
ENSG00000223711	-1.508802052	8.03E-05
NDUFS5	-1.50746972	3.65E-16
ADGRA3	-1.507383163	7.57E-08
NSA2	-1.503644775	7.40E-21
CNOT6L	-1.502649645	1.55E-10
ENSG00000271964	-1.500530368	1.68E-10
NRIP1	-1.499132289	5.03E-18
LDLRAD3	-1.498672715	6.05E-12
SEC14L1P1	-1.498467638	3.67E-09
TMSB4XP6	-1.49770882	7.23E-10
F13A1	-1.495575979	9.26E-08
ARHGAP21	-1.493802492	1.28E-39
MIF4GD-DT	-1.493744243	6.49E-13
RPSAP9	-1.490752117	5.95E-06
RPS29	-1.487544607	1.14E-19
PTMAP2	-1.486288844	1.95E-06
SPCS3-AS1	-1.48438614	3.57E-05
UQCRFS1P1	-1.483379083	1.39E-04
DIP2A-IT1	-1.483170118	1.96E-04
PEAR1	-1.482076546	2.62E-06
ENSG00000218426	-1.481255848	4.37E-20
ENSG00000227355	-1.478746693	1.63E-06
ENSG00000291112	-1.478606542	5.86E-08
RPL38	-1.477468403	8.90E-25
TLE1	-1.47599539	1.86E-13
RPS7	-1.475705344	1.46E-18
CNNM3-DT	-1.475272248	8.93E-06
ENSG00000275202	-1.474801317	5.51E-06
RPL18AP3	-1.474535657	8.42E-25
RSL24D1	-1.473231192	2.46E-15
RASAL2	-1.472263536	1.04E-05
ZNF354C	-1.471834991	1.97E-14
EEF1A1P6	-1.471120226	4.03E-14
LYPLAL1-DT	-1.469256388	2.51E-04
KLRK1-AS1	-1.469084047	1.26E-04
ZNF721	-1.468239873	1.53E-19
RPL6P27	-1.466985848	2.36E-12

TCAF1	-1.465560945	5.88E-06
KCTD3	-1.464812562	1.68E-12
MRPS31P5	-1.462247423	2.17E-05
BNIP1	-1.46087145	5.31E-21
TPT1P9	-1.460587292	1.92E-08
JMY	-1.459903946	1.87E-13
EIF4BP6	-1.459822546	1.23E-07
CHML	-1.458547247	1.14E-15
RPS15A	-1.457587243	1.09E-17
ENSG00000286089	-1.457546396	4.44E-04
RBM44	-1.455999488	3.05E-05
PLCL1	-1.455655712	9.43E-07
ENSG00000276517	-1.455256161	1.42E-06
LINC00989	-1.45213704	1.65E-08
WDR11-DT	-1.450262184	2.75E-09
RPL35	-1.448761753	1.13E-15
EEF1A1P5	-1.444435803	1.31E-21
PTS	-1.441082061	3.45E-11
PDGFA-DT	-1.440774279	3.53E-09
ENSG00000234961	-1.439528888	4.35E-04
RPL22L1	-1.439281759	3.35E-19
ENSG00000251867	-1.439036637	1.05E-06
RPS21	-1.433392538	2.17E-19
CHASERR	-1.433331876	6.54E-17
DUSP5	-1.432488667	1.35E-09
NDUFAF2	-1.432181639	2.12E-13
PTMAP4	-1.43100131	4.62E-09
RPL21	-1.430832174	7.03E-15
GPRIN3	-1.425859473	2.54E-13
SNORD46	-1.424794956	2.16E-04
BHLHE40	-1.424069041	7.29E-08
ENSG00000254682	-1.422787963	1.64E-09
ENSG00000240497	-1.420712917	9.34E-04
MIR590	-1.416393372	2.36E-04
RPL37A	-1.415528548	9.85E-25
SOX4	-1.415311097	3.69E-14
SNORD4A	-1.41334414	3.48E-09
KLF4	-1.411599807	8.85E-12
HTR7	-1.411052277	9.28E-06
CERNA1	-1.410758773	3.78E-06
RHOBTB1	-1.409980609	3.56E-05
RPL27	-1.409024515	3.57E-20
ARL4C	-1.407073705	1.03E-21
VDAC1P8	-1.405468305	4.29E-06
EPHA4	-1.404147801	1.72E-13
ZFP36L2	-1.403223729	9.98E-37
NGDN	-1.403167017	4.64E-19

DNTTIP2	-1.402302711	1.03E-15
SACS	-1.40205587	1.18E-15
SLC7A5	-1.402040325	2.20E-06
AFAP1	-1.4016088	1.32E-11
PANX1	-1.401311455	7.41E-12
EIF4BP3	-1.400297337	2.06E-09
APP	-1.398435923	3.02E-24
ENSG00000258181	-1.396501277	6.73E-04
NCBP2AS2	-1.396403167	8.49E-21
EIF3E	-1.39503518	8.92E-17
RANBP6	-1.394843499	6.76E-12
RPL24	-1.394115198	1.42E-16
ACAD11	-1.3934158	4.41E-07
HMGB3	-1.39005085	1.53E-06
COL19A1	-1.389697265	1.92E-05
EIF4A1	-1.389388748	1.65E-12
RPL10A	-1.3892598	1.15E-24
RPL21P28	-1.387540394	6.02E-11
LRP12	-1.38698245	7.51E-06
NRG1	-1.38584527	6.84E-04
ENSG00000286330	-1.385370704	1.24E-05
PMAIP1	-1.385223851	6.14E-08
TMEM170B	-1.381189272	1.72E-11
AGAP1	-1.380644863	7.23E-07
MTFP1	-1.379957679	2.17E-06
ENSG00000267469	-1.379951334	1.93E-04
SNHG25	-1.379869219	1.80E-13
WDR43	-1.379595979	1.47E-16
RPL7AP6	-1.378161857	4.83E-26
FSBP	-1.377994961	8.97E-06
KTN1	-1.377767477	5.43E-24
PNN	-1.376723433	2.57E-20
NACA	-1.373257444	9.79E-21
FAM169A	-1.370935995	1.08E-08
RPL32	-1.365530287	3.91E-15
MAST4	-1.363919265	3.62E-11
TGFBR3	-1.361882262	1.63E-08
ZBTB16	-1.361882207	2.25E-10
ADAMTS6	-1.36134323	1.47E-04
LCNL1	-1.359876553	3.87E-05
PPP1R15A	-1.359231294	5.89E-04
HNRNPA1	-1.359051374	2.72E-35
MTND4P12	-1.358785296	1.59E-06
CCT6B	-1.358616884	9.15E-06
KLRC1	-1.358477041	1.58E-06
MAP1A	-1.357963963	3.72E-07
DNAJB1	-1.357182145	7.39E-18

ZNF503	-1.355308216	1.21E-09
KLF12	-1.354804921	7.55E-17
RPL29P4	-1.352314783	1.03E-11
MEX3C	-1.352114944	3.86E-20
C1orf21	-1.351977028	6.29E-09
TSHZ1	-1.35108214	1.50E-31
NOP58	-1.350713102	1.20E-14
PRELID2	-1.350343733	4.97E-05
DNAJC2	-1.348924764	1.39E-15
TRPC1	-1.348686121	7.05E-06
TNNC2	-1.347358522	9.61E-06
BNC2	-1.345715062	2.42E-05
SNX25	-1.345352252	6.84E-19
RPS27	-1.344961002	3.60E-14
MLLT3	-1.344004247	5.12E-11
SREK1	-1.342968765	8.96E-17
CNTF	-1.342816096	2.90E-04
RPL13AP5	-1.342576918	8.70E-23
SNRPD2	-1.341910555	1.54E-19
PCDH9	-1.339853778	7.32E-04
N4BP2	-1.339787008	2.48E-08
RBAK	-1.339018257	2.31E-12
SFMBT2	-1.338512968	3.01E-15
RPS2P32	-1.338149749	2.26E-05
PBDC1	-1.336448737	1.35E-08
ENKUR	-1.3345408	5.38E-05
TOMM7	-1.334339412	9.36E-17
ENSG00000240376	-1.333976826	8.80E-06
CEP290	-1.333787675	1.29E-15
RLF	-1.333309254	3.27E-12
RAB30	-1.33046828	8.92E-10
ENSG00000288758	-1.329664252	4.80E-05
RPS20	-1.328688064	3.82E-16
RPL11	-1.328590467	1.35E-15
CSRNP1	-1.328458072	1.77E-07
ENSG00000288933	-1.328417948	1.68E-05
ZNF595	-1.328391598	2.19E-07
IFT57	-1.327530828	1.40E-15
TIGD1	-1.327201279	3.39E-16
ENSG00000288806	-1.326944939	9.40E-05
EIF2AK3-DT	-1.326499167	4.21E-09
ZBTB3	1.323637523	3.14E-18
ENSG00000276523	1.323652413	9.70E-06
IRF2	1.323754735	7.07E-22
TMEM169	1.324979017	8.24E-07
LINC00877	1.32578603	1.30E-14
MTG2	1.327526143	1.17E-31

ENSG00000279792	1.328052373	1.70E-04
VPS9D1	1.328825285	1.04E-11
ENSG00000258904	1.329035788	1.61E-04
RTN4R	1.329121031	4.17E-06
P2RX1	1.329148732	1.51E-10
PDLIM1P4	1.329408339	7.40E-05
SMCHD1	1.33087467	5.00E-14
MUC20-OT1	1.331951381	6.16E-06
PTPRN2	1.332933658	1.41E-14
ENSG00000268069	1.333241089	2.49E-06
PACSIN2	1.333717488	1.59E-42
ERV3-1	1.334124788	2.14E-12
RNASET2	1.33434442	2.62E-27
ARHGEF40	1.335307879	3.85E-11
FAM210B	1.337142283	1.48E-10
YIPF4	1.337171563	6.43E-32
ENSG00000289043	1.337625729	5.20E-09
DUT-AS1	1.338212879	4.08E-05
SEMA4B	1.338408437	2.75E-22
LINC00174	1.338977182	2.71E-22
UPB1	1.33975066	1.87E-04
ARHGAP25	1.340082901	5.17E-63
GPSM2	1.340156336	7.82E-13
ENSG00000279561	1.343783372	3.97E-04
ENSG00000278434	1.343952647	2.52E-05
ST8SIA4	1.344509565	8.11E-18
UNC13D	1.345457102	2.28E-24
SDCBP2	1.346472227	8.03E-09
SH2B2	1.347625086	2.26E-14
ANKRD42-DT	1.34974054	2.04E-10
DNLZ	1.350835032	1.97E-07
INSL3	1.351116691	4.88E-08
SLC38A5	1.351994577	1.40E-20
PLEKHO2	1.355581295	1.67E-27
LINC-PINT	1.356589789	8.65E-16
RMI1	1.356882781	2.58E-10
GPR25	1.356985405	2.60E-08
H2AC20	1.357077943	8.24E-07
SMIM38	1.357934467	7.86E-04
DOCK6	1.358186829	1.11E-09
PIK3R6	1.358395607	5.10E-12
CNIH3	1.358804665	1.18E-10
MOB3A	1.35915802	1.39E-33
TUBA1A	1.359981465	7.90E-21
ENSG00000227782	1.36096097	5.56E-05
SKAP2	1.361564888	3.84E-28
LINC01094	1.362761783	2.61E-07

ZSWIM3	1.363989072	1.83E-11
ENSG00000243155	1.364800211	5.16E-07
MBNL3	1.36594757	8.35E-11
RIGI	1.366427376	6.07E-05
ENSG00000268670	1.367679365	4.47E-07
ENSG00000260810	1.367709554	1.54E-04
SNX27	1.368276098	4.70E-26
TLR5	1.368384723	5.97E-11
CDC42	1.370245636	8.79E-18
ZNF818P	1.370544799	1.97E-06
HRH4	1.371269939	1.35E-05
KCNE3	1.371536377	2.57E-15
ENSG00000269982	1.373375409	6.72E-05
ENSG00000288953	1.373607841	3.74E-20
ENSG00000272356	1.373902121	2.95E-10
ENSG00000261582	1.374271287	4.29E-04
LTBP2	1.375732912	1.77E-09
TLR1	1.37921871	2.00E-19
AATBC	1.379315318	3.66E-06
ENSG00000235192	1.379395619	3.12E-06
CFLAR	1.379611208	1.04E-28
ENSG00000273855	1.38002862	3.71E-08
TMCC3	1.380907152	2.80E-08
LINC02631	1.382914114	1.09E-07
ENSG00000272918	1.384411414	3.95E-11
MYO7B	1.385439784	5.99E-08
FAM43A	1.386481707	2.50E-10
ENSG00000273230	1.388516128	1.91E-08
APTR	1.389232703	9.00E-15
C16orf54	1.38951894	1.25E-29
MIR25	1.391296977	1.22E-07
ABCG1	1.39472846	3.67E-17
PARS2	1.395092608	7.81E-05
CYTH4	1.39526392	5.59E-29
ARAP1	1.39732256	9.22E-24
ODF3B	1.397564519	2.51E-06
HDAC4-AS1	1.398503202	1.56E-15
RBCK1	1.398696598	1.99E-17
FUZ	1.400254061	1.23E-20
RRM2B	1.40038749	6.63E-25
RAB33B-AS1	1.400515122	1.64E-11
MAP7	1.40067302	3.67E-05
KIF27	1.400960508	6.01E-17
FRMD4A	1.401298092	4.50E-04
FMNL1-DT	1.401887692	5.93E-21
ENSG00000279759	1.403883457	9.49E-08
ENSG00000271533	1.404273242	1.79E-08

TEKTIP1	1.404445434	5.41E-12
DDX60L	1.404511057	8.55E-11
ENSG00000271971	1.404634453	4.09E-06
APBB1IP	1.405555105	1.19E-33
SLC16A5	1.407419371	4.05E-24
RILPL1	1.4078283	6.28E-12
FCHO2	1.407924576	2.24E-11
BACH1-IT1	1.408426068	1.98E-06
ENSG00000288792	1.408430915	8.13E-04
ENSG00000278576	1.409972464	3.45E-05
LINC00482	1.410020781	3.30E-08
ZNF470-DT	1.411509475	5.97E-04
HELZ2	1.411993726	7.31E-10
IFRD1	1.413054281	5.64E-10
SHKBP1	1.413332617	1.44E-22
KCNE5	1.41346677	1.51E-04
ENSG00000226266	1.415314356	2.23E-06
TSPAN2	1.416430781	7.64E-14
SELL	1.416689089	6.67E-25
CCDC121	1.418708694	1.81E-04
PRKD2	1.419139036	1.23E-26
FIGNL2	1.419284017	1.56E-10
IMPA2	1.41953934	6.23E-17
FCGRT	1.420187968	1.13E-30
ENSG00000267632	1.421133494	8.63E-06
SPOUT1	1.421482313	2.29E-07
FAR2	1.42363718	3.40E-15
ACTR3C	1.42493127	1.76E-09
ZNF117	1.425407747	1.17E-13
TMEM121B	1.426071462	7.72E-10
ENSG00000266980	1.426784864	7.44E-04
POLD4	1.428334949	1.07E-30
ANKRD13D	1.429477691	2.99E-25
EPOR	1.429921551	3.39E-23
PCBP3	1.430707201	1.61E-08
H3-3A	1.430846623	8.77E-38
CFP	1.432270022	1.09E-23
HIP1	1.432397845	1.39E-11
KYAT1	1.433436092	1.74E-05
ENSG00000275484	1.433835545	8.85E-04
PLB1	1.434336408	4.17E-11
NLRX1	1.436011599	6.14E-19
SVIL	1.436644839	3.87E-33
RAPGEFL1	1.436654286	5.97E-14
CLEC1A	1.43731208	1.18E-11
DBN1	1.43816486	7.04E-14
TMEM81	1.439820212	6.79E-10

CAMK2G	1.441181189	2.40E-31
CTBS	1.441295186	1.59E-27
ENSG00000277151	1.441766867	1.69E-07
PARP14	1.44255211	2.85E-09
CRISPLD2	1.442925441	2.30E-12
CCDC142	1.443471265	5.26E-14
JAK3	1.445076322	4.33E-16
RAB11FIP1	1.445919111	5.01E-29
TMEM86B	1.446469882	4.18E-20
ZNF547	1.446946629	7.12E-07
ASS1P1	1.44771593	1.00E-05
PIGX	1.44978747	2.50E-31
TCIRG1	1.450281596	8.25E-25
LINC00654	1.450607498	1.18E-08
SBNO2	1.451723122	4.63E-13
TALDO1	1.452221015	1.94E-42
ENSG00000267152	1.453160166	6.22E-07
BRCA1	1.454342295	3.29E-20
ENSG00000271857	1.454916933	6.67E-07
KLHL21	1.455078388	1.05E-23
GALNS	1.455659335	5.16E-20
MAST3	1.456419271	3.01E-17
BRICD5	1.45682241	1.36E-16
SIGLEC14	1.457243034	4.25E-10
ENSG00000279812	1.459269841	1.06E-04
ENSG00000276136	1.459546011	1.31E-21
ENSG00000217275	1.459649903	3.53E-05
IRF2-DT	1.459656213	9.81E-18
MARCHF8	1.459755329	9.15E-07
CASS4	1.461226144	1.17E-16
C5AR1	1.461787936	3.00E-14
ELL	1.463456765	6.47E-23
BACE1-AS	1.463842656	1.04E-08
OPLAH	1.464608744	6.02E-13
CR1	1.464950858	1.71E-11
TTN	1.465904753	1.19E-18
ENSG00000279162	1.466020073	3.35E-07
ADGRE2	1.466307686	7.01E-13
ENSG00000260060	1.466427248	1.71E-06
BSG-AS1	1.467234155	9.77E-08
CPPED1	1.467465134	2.34E-30
ENSG00000261123	1.467705906	2.44E-04
ENSG00000279108	1.467980586	2.51E-10
OSER1-DT	1.468600245	3.21E-10
ENSG00000289504	1.469189175	1.46E-10
ENSG00000233411	1.469643842	8.49E-07
GAB1	1.46967026	2.86E-22

PTP4A3	1.469851123	1.67E-04
NOXRED1	1.471520884	1.50E-07
ENSG00000267980	1.471767439	2.16E-07
LINC01232	1.471893569	1.94E-08
OAS1	1.472522087	6.04E-05
SULT1A3	1.473178728	2.18E-06
FGD3	1.474292746	3.99E-29
BAIAP3	1.474827459	1.38E-09
SORL1	1.474894384	7.63E-32
ENSG00000272555	1.475572275	4.29E-05
LINC02422	1.475763561	3.61E-19
CSF1	1.476135136	2.03E-14
CRELD1	1.478530815	6.18E-11
FAM53C	1.478641258	1.96E-28
ENSG00000280383	1.480184934	1.63E-06
SIGLEC10	1.480366529	3.24E-20
ENSG00000290958	1.480447363	3.31E-11
CDH13-AS2	1.482331234	4.19E-12
ENSG00000259704	1.483328745	3.19E-08
ENSG00000255946	1.484017936	4.04E-04
ARSA	1.488091261	1.24E-21
ELMO3	1.489717578	4.73E-17
TLR10	1.490634338	5.87E-09
PLA2G4B	1.491142809	3.62E-07
ENSG00000261799	1.491702449	1.78E-27
CKLF	1.492220292	5.56E-18
TG	1.49595579	2.20E-18
FBXL9P	1.496590108	5.29E-32
ZNF200	1.49674223	1.01E-28
ENSG00000205018	1.496854841	1.70E-07
ANPEP	1.497816982	1.73E-08
PBX2	1.498007629	8.74E-07
MTARC1	1.500473398	1.64E-13
PLXNC1	1.500545463	3.58E-27
ENSG00000215022	1.501245382	9.51E-16
ENSG00000291215	1.502133058	2.20E-12
ENSG00000269403	1.50379874	5.47E-05
SH3BP5L	1.505225703	2.67E-36
DTX2P1	1.505588384	6.30E-05
GAPT	1.506700071	1.07E-11
ENSG00000228107	1.507510302	4.40E-04
USP10	1.508072887	6.03E-42
ENSG00000266490	1.510058459	4.30E-06
PPP1R3D	1.512597721	3.27E-23
ENSG00000258674	1.513303878	7.15E-06
MOSPD2	1.516684284	7.34E-14
WAS	1.516727176	1.50E-30

ENSG00000277507	1.518169925	6.52E-05
TRAJ35	1.518570095	9.92E-04
XAF1	1.518820536	4.33E-05
SOD2	1.520822246	5.34E-07
LINC00957	1.520962077	3.04E-19
H3P6	1.521956728	5.34E-12
LRRC51	1.52218594	4.31E-11
ENSG00000257221	1.522284838	1.19E-08
ENSG00000279035	1.523156611	2.69E-04
DTX2	1.523663975	4.62E-24
CAMKK1	1.524337032	2.99E-15
ENSG00000289883	1.525771815	7.27E-04
UBN1	1.525964616	6.16E-32
ENSG00000272084	1.526105365	1.71E-09
ENSG00000286965	1.526260031	5.41E-04
TREM1	1.527008447	1.97E-13
FAM8A1	1.52703092	2.27E-18
ENSG00000277767	1.527711968	3.87E-06
ENSG00000280332	1.528569866	1.70E-24
LINC01410	1.530692892	1.81E-07
TMEM120A	1.532211835	5.87E-41
PILRA	1.534128587	1.90E-24
YJEFN3	1.536156706	1.74E-11
FLVCR1-DT	1.536192037	3.20E-06
PARP9	1.537246845	7.03E-11
FANCA	1.538031482	1.11E-09
USP34-DT	1.538039353	2.57E-17
CSAD	1.539064382	1.68E-21
PLEKHG3	1.539443734	2.36E-16
MPPE1	1.539651473	4.49E-63
OLIG1	1.540781009	4.65E-12
ENSG00000228434	1.541988915	6.17E-12
LIN37	1.544049116	5.30E-21
LY96	1.54410228	9.01E-16
EFCAB8	1.545995352	5.26E-13
LINC00528	1.546502668	1.18E-28
NOTCH1	1.547756044	3.57E-22
MRPL20-DT	1.548455887	1.19E-12
DSC2	1.548978996	3.06E-08
ZNNT1	1.551262481	2.60E-09
ENSG00000276900	1.55188073	1.96E-14
ENSG00000270574	1.553118394	5.04E-12
ENSG00000286796	1.555155194	1.21E-08
IGF2R	1.55607181	1.81E-22
B3GNT9	1.556137375	3.82E-19
SLC2A1-DT	1.557557062	8.35E-07
ENSG00000289042	1.557779568	2.11E-10

DYNLT4	1.55809029	2.29E-04
ENSG00000272379	1.5584445	6.86E-11
ADAMTS7P1	1.558899391	9.06E-07
RPS6KA5	1.559214856	2.74E-36
TRAJ34	1.56002105	6.32E-04
ENSG00000272112	1.560073877	8.43E-11
ENSG00000278112	1.560143803	4.04E-05
ENSG00000286817	1.560551144	7.17E-05
EEPD1	1.562819017	7.10E-28
PGS1	1.563450742	4.99E-22
PINK1	1.563622999	2.07E-31
ENSG00000291144	1.565048295	2.88E-14
EPHB4	1.565548168	4.63E-23
EFCAB12	1.568473601	4.93E-13
ENSG00000270277	1.569926769	7.31E-09
TRAJ28	1.570726277	6.22E-04
ENSG00000279203	1.572001053	1.13E-08
NUP50-DT	1.572433715	4.24E-18
ENSG00000260111	1.577587243	9.42E-06
NTAN1P2	1.579930442	1.24E-04
ZBP1	1.5802608	4.12E-10
ENSG00000233547	1.581196967	4.03E-05
NQO2	1.581595682	4.20E-14
OPRL1	1.581688009	9.64E-09
MIR3176	1.583451234	5.42E-07
ENSG00000291232	1.584484514	1.85E-04
VAMP1-AS1	1.584860451	7.53E-13
SNAI3-AS1	1.584973425	1.22E-18
SHROOM4	1.588273347	2.33E-04
FXYD2	1.588598658	2.61E-04
CYBC1	1.588850997	3.51E-35
NBEAL2	1.592305586	8.59E-18
TRAJ20	1.592370036	8.25E-04
SRPK1	1.59351569	5.25E-29
IMPDH1	1.594215309	5.44E-31
RGL4	1.594275605	3.92E-22
ADIPOR1	1.596534235	8.34E-16
STK40	1.599505703	5.54E-32
ACTN1	1.600483671	1.75E-30
ENSG00000288838	1.601447903	3.43E-10
ENSG00000273619	1.602790416	6.02E-11
PYGM	1.602990449	5.84E-26
XXYLT1-AS2	1.603345992	8.33E-04
ENSG00000279570	1.603631674	4.00E-07
LINC02970	1.605368969	1.34E-06
ZNF746	1.606174096	4.11E-28
ENSG00000288819	1.607234826	1.38E-06

PDZK1IP1	1.607503717	1.20E-05
APOL1	1.607642241	3.28E-10
HSH2D	1.608564675	2.53E-23
HEXIM2-AS1	1.611461713	1.27E-51
ENSG00000273449	1.61195806	1.86E-07
MTX1	1.612021839	1.08E-28
S1PR4	1.612216715	2.18E-47
ENSG00000285796	1.612396118	4.83E-32
HLA-C	1.613817905	9.43E-10
DMTN	1.615362307	1.87E-06
ENSG00000262370	1.61590505	2.03E-21
CHST15	1.619634047	1.81E-29
NLGN3	1.620120223	1.79E-18
B3GNTL1	1.621592956	3.26E-20
ENSG00000288061	1.621788767	3.54E-17
APOBR	1.621968941	4.58E-27
ENSG00000279344	1.628062805	4.06E-04
SSPN	1.629527649	4.88E-06
ASLP1	1.630555237	1.40E-06
AIM2	1.632273281	8.61E-07
LITAF	1.632284641	6.67E-24
RAB19	1.633012259	5.19E-07
ENSG00000288886	1.633657025	3.21E-08
NUAK2	1.635652735	1.44E-34
MIR3677HG	1.636672712	2.05E-08
ENSG00000276791	1.638579441	9.27E-15
RNF149	1.638890361	5.22E-26
ENSG00000287100	1.639286898	2.67E-07
ENSG00000274341	1.639516631	1.37E-08
SNORD17	1.641063769	4.52E-06
IL6R	1.641132029	8.35E-53
ENSG00000268743	1.642467721	9.68E-05
ENSG00000249863	1.643013596	9.42E-07
SECTM1	1.644634154	3.68E-15
ENSG00000260563	1.645355068	4.78E-25
SMIM24	1.646481841	1.74E-04
PTGDR2	1.649394739	1.06E-10
PGAM2	1.649956259	9.29E-08
WWC3	1.651089478	6.11E-35
LINC01004	1.6511213	8.20E-37
ENSG00000277218	1.651738522	1.10E-04
NCF2	1.651905571	9.22E-35
ENSG00000270120	1.654681599	3.90E-06
BORCS8	1.65497534	2.18E-29
MIR4312	1.655430195	3.50E-04
SLC6A6	1.656376513	1.50E-33
MIR106B	1.659467153	1.19E-04

ENSG00000278963	1.659549515	2.82E-08
ENSG00000288887	1.661169252	1.49E-06
ENSG00000248367	1.661417154	5.37E-27
COL9A3	1.661612101	2.74E-08
F11R	1.664688214	2.52E-76
ENSG00000273154	1.665122202	9.39E-04
TRAPPC14	1.665423427	1.17E-30
ST3GAL4	1.665437712	2.71E-25
SORL1-AS1	1.666455067	1.08E-08
TRAJ19	1.667321682	2.09E-04
SLC11A1	1.667478485	2.80E-14
VASP	1.669683719	8.04E-39
TCF3P1	1.670027311	3.19E-05
ENSG00000236194	1.671257164	2.17E-09
TRIM25	1.671695399	1.46E-19
ABHD5	1.673013317	2.11E-29
ENSG00000264083	1.674222557	3.05E-04
ENSG00000272345	1.675450819	3.02E-14
DEF8	1.675719248	5.30E-44
NR6A1	1.676770114	1.68E-18
ENSG00000275437	1.677024255	8.49E-16
ENSG00000266718	1.677854813	7.26E-05
TTC26	1.679251097	1.66E-07
ENSG00000268362	1.679305686	4.94E-21
TAPBP	1.679460499	2.03E-07
C17orf99	1.679898544	3.39E-04
FAR1-IT1	1.680609608	1.26E-04
ENSG00000291015	1.6825822	7.50E-23
ENSG00000279549	1.682617948	6.17E-10
SLC22A18	1.684134475	1.31E-13
CCDC194	1.684368466	4.49E-10
CACNG6	1.684494373	7.50E-09
LAMP2	1.685523442	2.02E-50
ENSG00000290563	1.68724616	8.11E-08
CBLL1-AS1	1.687965754	1.95E-10
AP1M2	1.688094961	3.46E-07
MAPK13	1.688393366	1.63E-31
ENSG00000289425	1.689045421	5.08E-05
NAMPT	1.690552273	3.34E-05
RGS14	1.690569094	1.62E-28
ENSG00000286264	1.691409059	3.60E-11
KIAA1614-AS1	1.691724535	1.18E-09
DGLUCY	1.69186598	1.50E-32
TMED2-DT	1.694171328	2.38E-13
SSH3	1.694425798	8.17E-30
LRRC46	1.694918756	5.97E-14
PLPPR2	1.695201579	1.21E-25

NPIPB7	1.695769105	6.86E-07
LINC00899	1.696389315	1.48E-07
UPK3B	1.696502588	1.74E-08
NDEL1	1.697027426	7.33E-36
ENSG00000280007	1.697045786	2.23E-05
ENSG00000214773	1.697275879	1.20E-04
ENSG00000275180	1.697314806	1.31E-14
CCDC125	1.697501954	7.26E-10
MIR3671	1.697780201	5.84E-10
ICAM3	1.699190994	8.26E-55
ADAM8	1.69984943	4.05E-26
SLC16A13	1.700845507	1.83E-10
CLEC9A	1.701666692	1.14E-07
MTND5P14	1.705216906	3.56E-04
ENSG00000289912	1.705347344	2.93E-05
PTPN23-DT	1.70572525	3.54E-14
ENSG00000289691	1.707097135	1.14E-21
UBXN2B	1.707917821	1.67E-49
ENSG00000240207	1.708109273	4.30E-04
KBTBD7	1.708837596	4.22E-19
HSD3B7	1.709630769	3.45E-12
KRT18P5	1.711493582	6.95E-05
ENSG00000261864	1.711893694	5.92E-04
ENSG00000267436	1.712932699	2.11E-17
OLFM2	1.714291292	5.63E-10
DCST1	1.714307331	4.03E-05
CCDC17	1.717585062	5.42E-22
CTCF-DT	1.718338985	1.26E-08
NHERF4	1.718465661	1.16E-04
ENSG00000288612	1.718813482	9.49E-12
NT5DC4	1.720172287	4.77E-04
GADD45G	1.720485954	1.87E-08
CLC	1.721729427	3.15E-14
CABP1	1.722679164	7.24E-04
DRC7	1.72483626	3.27E-06
TMEM240	1.727807067	2.31E-06
KATNBL1	1.729457679	2.34E-28
IKBIP	1.730344865	3.33E-26
LINC00921	1.731321242	8.93E-36
GHRLOS	1.734465914	1.05E-08
GBP5	1.73646711	1.09E-04
H2BC5	1.737742475	7.34E-09
ABCA7	1.737760302	6.14E-31
DPEP2	1.738261551	3.17E-40
ENSG00000255142	1.739459546	1.04E-06
LAMP3	1.740944602	8.18E-04
ENSG00000287975	1.741209547	1.26E-06

ENSG00000289935	1.742740998	2.10E-05
ENSG00000278600	1.744633304	2.48E-43
HCK	1.745205641	3.44E-34
ENSG00000280088	1.745825411	7.47E-40
ENSG00000260160	1.747036852	9.64E-14
LPCAT2	1.748197355	4.46E-27
ENSG00000280129	1.749398465	8.07E-06
IL1RAP	1.749839325	9.55E-20
FCGR2B	1.751647395	1.91E-14
CADM4	1.752600637	2.55E-22
ENSG00000267263	1.752894401	9.55E-09
TNFSF10	1.753251493	4.87E-14
KCNE1	1.753655277	1.40E-11
S100A11	1.753884535	1.31E-34
DHRS12	1.754411274	8.54E-23
PRDM8-AS1	1.754915232	3.81E-04
CC2D2B	1.755063215	4.11E-10
DHRS13	1.755138188	1.45E-31
JAML	1.757892529	9.86E-49
ABCB6	1.75910974	5.23E-09
ENSG00000279357	1.760366069	6.18E-10
RNU6-611P	1.761868651	1.42E-06
LIN7A	1.762052417	3.94E-15
USP7-AS1	1.762097844	9.29E-07
HEATR6-DT	1.762255103	2.49E-04
CFAP251	1.762601037	3.25E-11
ATP6V0D1-DT	1.764463596	1.43E-16
RFX2	1.765370005	2.74E-17
ENSG00000264666	1.766636258	7.25E-04
ENSG00000290040	1.768299735	4.41E-04
NATD1	1.774437131	1.70E-21
ARHGEF17	1.774620034	2.37E-05
PROC	1.775028328	5.35E-07
CLUHP3	1.775349336	3.15E-04
ENSG00000289155	1.776365294	8.64E-09
ENSG00000289082	1.776751751	4.25E-07
ENSG00000280106	1.776757927	1.52E-09
LINC01134	1.77791258	1.52E-08
AMT	1.779004598	1.00E-04
ENSG00000281468	1.779277756	1.97E-05
LINC02193	1.779283656	8.04E-11
ENSG00000285886	1.783566548	6.79E-07
ENSG00000269318	1.784152958	6.77E-07
SLC12A9	1.784510243	1.74E-38
ENSG00000229591	1.784542148	6.67E-09
ENSG00000271009	1.785530412	4.11E-07
DNAJC8P2	1.785777268	3.10E-04

ADM-DT	1.787418568	6.76E-04
XKR8	1.788026429	1.23E-57
TMEM234	1.78809394	5.14E-49
ENSG00000242324	1.789190778	2.29E-06
ALOX5AP	1.79151039	4.00E-34
ENSG00000250644	1.792895141	2.54E-05
WDFY3	1.792965473	1.41E-25
ENSG00000242539	1.792974471	3.71E-07
SULT1B1	1.793812945	1.57E-14
ITM2B	1.795367761	1.33E-55
PCBP1-AS1	1.797262902	7.70E-44
ENSG00000279841	1.797742722	2.89E-09
ATP5MGP1	1.798713604	6.83E-05
ENSG00000232450	1.799581419	3.17E-05
ZMYND10	1.800254471	4.26E-11
ECI1-AS1	1.804105754	8.07E-05
ENSG00000289171	1.804605573	1.93E-04
BORCS8-MEF2B	1.80544153	1.81E-04
PHC2	1.806797005	6.47E-24
ENSG00000269463	1.807512525	7.30E-07
ENSG00000289055	1.808379908	1.36E-07
ARL11	1.80958087	2.23E-25
TMEM132E	1.810794962	9.49E-04
C3orf62	1.81309924	7.37E-33
ENSG00000272512	1.81325782	9.65E-04
ENSG00000235111	1.814845632	3.04E-21
SIRPB1	1.816625994	1.53E-32
MIR4489	1.816884387	6.26E-10
ENSG00000262312	1.817433861	4.86E-04
ENSG00000258317	1.817530929	2.00E-07
TRANK1	1.820102408	3.88E-22
MIR503HG	1.820626262	7.93E-11
LETM2	1.820963782	1.83E-16
ENSG00000223834	1.821027832	8.54E-04
NBPF19	1.828584911	6.79E-13
CAP2P1	1.828713312	3.48E-04
ENSG00000288880	1.830087802	1.92E-10
NLRP12	1.830171651	5.79E-19
ENSG00000280604	1.831942555	3.31E-05
TAFA2	1.832018295	6.20E-15
GARIN1A	1.832493374	6.24E-21
TPST1	1.83274937	1.67E-10
ENSG00000258082	1.835530175	1.21E-11
RN7SL535P	1.836800029	2.05E-04
SNHG22	1.838079989	7.61E-13
TRAJ24	1.838729644	2.96E-05
ENSG00000261659	1.838849687	1.95E-15

ENSG00000261386	1.838907041	1.59E-15
CRACDL	1.840179197	1.38E-12
DOCK8-AS1	1.841292737	5.24E-15
PABPN1L	1.841422711	9.29E-07
ARHGAP27	1.841425102	4.03E-09
PPIAP29	1.841606008	1.72E-04
MX1	1.84204214	4.98E-05
RN7SL172P	1.842523452	4.95E-11
ISG15	1.846021378	1.12E-04
ENSG00000251259	1.847560175	7.16E-11
ENSG00000274184	1.848320516	3.66E-04
ENSG00000289317	1.848369823	6.27E-05
RTN2	1.849111812	3.93E-25
ENSG00000237101	1.849126641	7.63E-04
TNFAIP8L2	1.851136703	3.60E-26
ENSG00000257475	1.85115557	1.80E-04
UBE2C	1.851791608	1.98E-09
RNASEL	1.853724077	1.82E-30
ENSG00000240401	1.857410915	3.38E-11
SCARNA9	1.857482574	7.15E-09
F12	1.857512264	4.91E-20
ENSG00000278467	1.858354524	2.03E-04
PPP1R3B	1.859209835	1.41E-07
ENSG00000261430	1.860031131	3.79E-04
ENSG00000290791	1.860461977	1.27E-28
RMI2	1.863802299	7.44E-04
ENSG00000228463	1.864194146	4.72E-07
LRP10	1.868161953	3.85E-50
ENSG00000224950	1.869068606	1.10E-17
SEC14L1	1.869863283	3.30E-25
ENSG00000259926	1.870641595	3.04E-05
ANKRD34B	1.871168762	2.61E-04
ENSG00000264188	1.8718844	8.91E-06
UFL1-AS1	1.872668935	5.07E-05
MEFV	1.874619143	8.03E-23
PNPLA1	1.87480998	9.56E-20
PRNCR1	1.876903002	3.20E-06
SOWAHD	1.878183373	3.71E-19
ENSG00000273189	1.878507532	8.20E-09
HSP90AB4P	1.879870452	3.55E-04
WFIKKN1	1.880497175	2.00E-20
MX2	1.880535911	1.82E-24
LINC02988	1.880643099	1.09E-19
PTAFR	1.881103754	3.98E-39
HERC5	1.881844574	6.78E-05
ENSG00000284685	1.882752446	4.94E-21
ENSG00000277763	1.882753687	1.63E-15

ENSG00000225411	1.884478837	1.44E-05
FAM174A	1.885643048	2.12E-21
RPS6KB2-AS1	1.886690467	1.45E-12
ENSG00000288156	1.887257783	1.65E-66
SLC22A13	1.888433962	3.80E-04
H2BC6	1.889140157	4.62E-09
TMEM30BP1	1.891913927	1.62E-10
PAGE2B	1.892813622	5.75E-05
ENSG00000279693	1.893105685	2.76E-08
ENSG00000266126	1.893149935	3.51E-08
LINC02913	1.893466562	7.21E-07
UBE2D1	1.894194419	4.58E-19
ENSG00000272911	1.89460024	5.05E-07
ENSG00000278000	1.901846983	3.26E-07
ENSG00000279140	1.901953452	1.29E-08
ENSG00000278869	1.902145691	7.92E-14
ENSG00000291174	1.903144057	5.90E-11
TRIM9	1.903171521	6.17E-08
ENSG00000281100	1.903417932	1.66E-14
LINC03073	1.905189101	2.39E-08
KIAA0825	1.907429573	7.21E-19
PA2G4P4	1.907515113	5.21E-04
STX5-DT	1.907745849	1.35E-07
ENSG00000278979	1.908390704	6.88E-04
MXD3	1.90890295	1.60E-45
LINC01819	1.909387328	3.95E-12
ENSG00000279330	1.909793773	3.52E-28
RNF216-IT1	1.91045454	5.02E-07
ATP2A1	1.91173616	1.12E-14
TMOD1	1.913485241	1.35E-09
SH3D21	1.913553326	4.35E-29
SLC16A3	1.914733999	6.30E-49
TP53I11	1.916756531	2.95E-26
PIP4P2	1.916892043	2.87E-45
FAM200C	1.917334543	1.93E-05
GYPC	1.918154887	3.05E-17
LILRA6	1.918817213	2.20E-09
H2BC7	1.918835183	1.13E-05
ENSG00000255089	1.919188018	9.85E-05
ENSG00000275162	1.920401656	2.08E-08
FCGR1A	1.920809242	7.45E-06
UBE2CP5	1.924148262	2.64E-34
IRF7	1.92492266	6.19E-11
ENSG00000287868	1.92612263	1.04E-11
ENSG00000271806	1.927163058	6.10E-04
OTOF	1.92894518	2.23E-04
MBD6	1.931865029	3.04E-32

SPNS2	1.931876903	8.83E-14
ENSG00000261798	1.93361022	7.17E-04
OASL	1.934101751	1.32E-07
PELATON	1.936364059	3.68E-22
ENSG00000267288	1.939459208	3.85E-20
MYO1F	1.940864488	2.13E-42
AP3B2	1.942659835	9.83E-04
RN7SL328P	1.945772632	6.92E-07
ACOX1	1.94590063	6.07E-80
VMP1	1.946466193	2.80E-47
ENSG00000257094	1.950443337	5.11E-05
CDA	1.951437382	2.59E-24
ENSG00000250696	1.952663938	3.80E-06
TYRO3P	1.953423784	2.51E-06
XPO6	1.954458736	3.53E-46
ENSG00000279073	1.95519194	3.16E-10
PISD	1.95731292	6.79E-57
MEF2B	1.958043987	1.82E-15
ST20-MTHFS	1.958808274	6.19E-08
R3HDM4	1.959372286	3.81E-36
RPL13AP26	1.961348656	2.46E-24
LIME1	1.961603249	1.90E-23
CCPG1	1.96470395	4.08E-29
STRADB	1.964995318	1.16E-11
DOK3	1.968378142	9.88E-28
RPL4P7	1.96849304	1.33E-06
ST6GALNAC6	1.969012023	5.50E-15
TNS1	1.969710138	3.88E-06
TRIM69	1.975267862	1.16E-12
TMEM91	1.975868753	1.08E-53
KDM6B	1.976003175	1.22E-24
ARG1	1.976283154	2.22E-04
MTCO2P11	1.977169241	1.74E-06
ENSG00000273110	1.977620288	2.61E-04
ENSG00000278002	1.979076302	1.28E-06
NFAM1	1.980245974	2.24E-41
ENSG00000284977	1.981092671	1.15E-11
RAB3D	1.982394066	1.06E-47
ENSG00000284820	1.982694755	2.25E-04
KLHDC8B	1.984295181	1.09E-25
FAM3D	1.986095076	5.64E-04
ENSG00000273363	1.98711047	5.68E-05
YPEL3	1.987607344	1.27E-45
ENSG00000251034	1.988407706	1.75E-04
LCN10	1.990294054	8.52E-07
KCNQ1OT1	1.990823057	1.80E-18
ENSG00000288604	1.991718402	3.26E-12

RN7SL381P	1.992699273	7.07E-09
CALML4	1.995676351	4.31E-08
MED25	1.996819024	2.43E-43
DOK4	1.997566689	3.53E-31
TRIM21	1.999500732	4.99E-42
DOCK5	1.999803165	1.80E-34
KISS1R	2.00134354	7.10E-05
CEBPE	2.002239395	1.66E-11
ABCA1	2.003096969	6.04E-15
HORMAD1	2.003655275	7.34E-08
TNFRSF1A	2.004735239	1.60E-45
ENSG00000289564	2.004946442	1.14E-06
ENSG00000275857	2.006508527	3.37E-07
ENSG00000278668	2.007207901	1.40E-09
FGF22	2.008934976	4.60E-08
ENSG00000289142	2.009715954	3.88E-04
RNU6-892P	2.010051469	2.01E-08
ORM2	2.012017664	5.34E-04
ZNF564	2.012110288	8.21E-12
LRRK2	2.012331106	1.82E-29
RAB36	2.012399232	3.36E-25
TRAJ33	2.012637485	2.50E-05
SCUBE3-AS1	2.013155537	1.69E-17
AMH	2.013572204	9.86E-07
RAB24	2.014808139	1.82E-30
ENSG00000291131	2.019777844	3.69E-19
CACNA1D	2.019903554	3.09E-09
PLGLB2	2.02014902	9.49E-05
SLFNL1	2.020539025	2.77E-06
C9orf163	2.021000424	8.19E-05
MPZL3	2.023771541	4.36E-30
NUDT16-DT	2.023921413	1.73E-18
ENSG00000280402	2.024573855	3.02E-12
PRMT5-DT	2.024733783	1.74E-08
COL7A1	2.028443595	8.09E-16
SLC25A39	2.031397327	3.06E-14
ENSG00000213600	2.034827399	9.69E-15
SIRPB2	2.037844997	2.55E-27
ENSG00000277566	2.038984949	3.09E-05
MFSD6L	2.039101442	1.05E-09
RNU4-62P	2.039186675	6.17E-06
GLT1D1	2.040120643	1.50E-30
NRBF2	2.041439732	3.24E-32
KLHDC7B-DT	2.043797612	4.34E-07
LINC02975	2.04381641	1.21E-14
UBR5-DT	2.045225876	7.43E-42
ENSG00000272183	2.0478872	4.97E-04

TRAJ32	2.049771403	5.22E-07
ENSG00000290018	2.052211199	5.76E-17
LINC01220	2.053606743	8.10E-05
ENSG00000274902	2.055259692	6.74E-08
ENSG00000230149	2.058197365	6.84E-16
WLS	2.058586246	3.45E-27
MIR6753	2.064064596	6.34E-15
ENSG00000288995	2.064166212	1.38E-05
ENSG00000277548	2.065579192	3.90E-08
SAP30L-AS1	2.06558596	8.16E-26
ENSG00000279744	2.065629894	7.55E-18
RPS12P27	2.06594703	5.23E-07
PIK3CD-AS1	2.068799752	4.67E-14
ENSG00000277715	2.069647181	2.56E-20
ENSG00000269892	2.070446607	1.19E-24
RN7SKP269	2.075838901	2.68E-04
ENSG00000290126	2.07845628	2.36E-09
PRR7-AS1	2.079972601	7.09E-10
SNORA73B	2.083664223	3.81E-16
LILRB3	2.084041826	1.49E-24
ENSG00000232828	2.08733334	3.06E-06
HTATSF1P2	2.089305343	4.49E-07
MIR1273H	2.093152909	6.46E-04
ENSG00000276248	2.097472486	5.38E-07
NHSL2	2.098800974	2.87E-31
GPR21	2.099093848	8.64E-05
ENSG00000289437	2.100727806	8.17E-06
GPR27	2.100902143	1.25E-21
ENSG00000289439	2.101961169	1.64E-05
ENSG00000252690	2.104597754	5.59E-15
TCP10L	2.107118576	3.63E-04
ENSG00000287611	2.107242032	3.09E-04
S100P	2.109050208	1.53E-04
PPCDC	2.110084242	9.21E-66
TBC1D3L	2.110777358	1.53E-05
APOL2	2.113806591	3.10E-22
FRAT1	2.115921521	2.05E-68
ITGAD	2.116511579	4.98E-10
ENSG00000283045	2.117055392	1.36E-04
ENSG00000279981	2.120386938	5.01E-05
ENSG00000205537	2.123349571	3.35E-07
ENSG00000285596	2.125600351	1.20E-24
AOAH-IT1	2.126911592	1.02E-10
PFKFB4	2.130901696	6.03E-42
ALPK3	2.131590372	3.64E-16
ENSG00000283602	2.132987968	6.36E-15
ENSG00000279696	2.134537904	4.89E-16

GSK3B-DT	2.135419418	1.80E-14
ENSG00000279653	2.13572606	5.73E-04
ENSG00000279591	2.137144248	2.25E-04
ENSG00000273243	2.137916441	2.06E-10
ENSG00000280388	2.138645876	1.81E-18
ENSG00000225300	2.138925174	2.92E-06
DCAF12	2.139129401	8.47E-13
CASP5	2.139221085	4.85E-11
RPGRIP1	2.141160157	1.03E-28
LINC02917	2.144447347	8.58E-04
ENSG00000279741	2.145696349	1.36E-13
ENSG00000261136	2.147562973	1.68E-19
ENSG00000235834	2.148057912	3.23E-07
ENSG00000274213	2.15151339	2.59E-13
ENSG00000268401	2.151521677	1.78E-06
ENSG00000279722	2.151838231	1.83E-11
GBP1	2.152186683	6.69E-08
ENSG00000279913	2.153738579	2.23E-04
ENSG00000275709	2.156023012	3.98E-05
GDPD3	2.157519756	2.40E-26
TSC22D1-AS1	2.159606296	1.99E-11
TRAJ38	2.159631415	1.89E-08
NLRP9P1	2.160412041	4.21E-08
PYGL	2.16175546	4.29E-47
FUT7	2.162318281	8.15E-45
ENSG00000287665	2.162892501	1.31E-11
ENSG00000227598	2.163086792	1.00E-10
LINC02656	2.1632813	2.74E-15
NALT1	2.164876221	6.52E-16
MYL6B-AS1	2.165260242	4.80E-05
ENSG00000284882	2.167565326	3.21E-20
RARA-AS1	2.168245068	1.97E-42
DENND3	2.168254949	1.50E-33
ENSG00000235189	2.168407979	6.61E-04
BTBD6P1	2.168704702	2.53E-06
ENSG00000290588	2.168811663	5.01E-04
GATA1	2.1694086	5.45E-11
OR2W3	2.172118818	2.13E-05
P2RY14	2.172130189	1.55E-11
PCF11-AS1	2.172639902	8.17E-23
ENSG00000291188	2.173347591	6.17E-18
BCL6	2.178170302	4.65E-17
ADM	2.178807627	6.78E-06
CAPN10-DT	2.17898946	4.57E-57
ENSG00000280003	2.179292505	4.46E-13
ENSG00000279328	2.181345754	6.02E-16
ENSG00000273444	2.182473139	7.58E-04

MIR499B	2.183034402	3.63E-04
LINC02352	2.183648454	6.36E-08
DNAJC3-DT	2.183714695	1.23E-15
ENSG00000229808	2.184420531	1.51E-16
NADK	2.186039461	2.92E-49
FBXO39	2.189020235	5.33E-04
RNA5SP352	2.194316021	5.68E-06
NPL	2.195885619	4.52E-41
GNA15-DT	2.197176373	1.63E-06
ENSG00000288906	2.197260218	2.93E-09
NRADDP	2.197511496	1.03E-11
ENSG00000203327	2.197708589	7.38E-05
FKBP8	2.197984469	4.71E-28
ENSG00000273367	2.202493105	1.46E-04
UPF3AP3	2.204907425	5.24E-06
ENSG00000260517	2.205205001	5.92E-17
ENSG00000261544	2.206062054	1.77E-05
ARHGAP9	2.207274465	2.63E-48
ENSG00000287185	2.209102394	6.88E-05
ENSG00000289514	2.209585445	3.77E-04
COP1-DT	2.21015018	3.85E-05
SLC22A14	2.212694763	2.11E-04
PNMA6A	2.213423485	2.66E-10
QPCT	2.216757905	5.22E-36
RAB3IL1	2.218866027	1.09E-05
ENSG00000260293	2.218893345	4.61E-07
ENSG00000258875	2.219478212	5.74E-21
RPS3AP18	2.220315562	2.39E-12
ENSG00000289421	2.220877075	3.36E-05
RPAP3-DT	2.221541053	4.21E-28
RASGRP4	2.228921049	3.71E-37
COL18A1	2.231349864	1.13E-16
NLRP6	2.231447485	6.96E-21
ENSG00000279632	2.23202917	2.69E-06
PACERR	2.23266794	2.31E-05
WDFY3-AS2	2.232800462	8.58E-07
NABP1	2.23552026	2.36E-37
ENSG00000276916	2.236097237	8.34E-07
PAN3-AS1	2.236531367	9.63E-26
RSPH9	2.238429383	5.77E-06
MYBPC3	2.238853588	5.17E-22
BGLAP	2.238881166	4.00E-16
ENSG00000272417	2.239936146	3.38E-05
RPS12P26	2.240544545	1.42E-08
KLF2-DT	2.242717414	5.25E-04
RN7SL812P	2.2428515	7.45E-04
MIR26B	2.243101553	5.81E-06

BACH1-IT2	2.247628189	4.66E-15
MPZ	2.250179667	1.18E-42
PGLYRP1	2.250224592	1.95E-08
CFAP418-AS1	2.250428487	5.25E-06
ADAM20	2.253171492	4.95E-07
DDX10P1	2.256914046	1.03E-05
ENSG00000276471	2.259328554	7.35E-05
ENSG00000230107	2.259507317	3.83E-05
H1-3	2.260187746	1.36E-06
CEP63	2.261021783	9.24E-64
ENSG00000279236	2.261686999	1.01E-06
SULT1A2	2.262326736	2.62E-09
ENSG00000258749	2.263722678	3.45E-04
CMTM1	2.267042265	5.33E-13
ITPKB-IT1	2.267116229	1.77E-24
ENSG00000273112	2.269429526	7.33E-13
ENSG00000286511	2.271151364	8.28E-04
ENSG00000274979	2.271303123	8.45E-09
ENSG00000273017	2.27174703	3.17E-09
ENSG00000289347	2.272595031	2.18E-15
PPP1R12B	2.274394066	1.53E-60
TRAPPC5	2.27543437	6.49E-26
ENSG00000267430	2.277244325	1.42E-04
RN7SKP9	2.279218266	2.68E-05
LRWD1	2.283576916	8.92E-40
UHRF1BP1L-DT	2.284062761	1.97E-04
NOL12	2.28589606	1.99E-50
GNB1-DT	2.286830591	5.38E-08
TMEM253	2.288793626	6.55E-05
ENSG00000265713	2.289461355	9.36E-06
ENSG00000272735	2.291796731	3.18E-04
RN7SL566P	2.292957837	1.43E-06
ENSG00000277383	2.295663164	1.73E-14
CCDC153	2.296419057	3.60E-36
ENSG00000288703	2.298157031	8.31E-05
MPZL1	2.299917088	3.20E-68
F2RL1	2.301506175	1.31E-31
ITGAX	2.302097705	9.69E-35
ENSG00000249309	2.302342127	4.49E-04
ATP2C2	2.304454038	7.49E-04
LINC02680	2.304561724	1.74E-12
ENSG00000289391	2.305892867	2.61E-04
GHRL	2.308325847	1.11E-46
NIBAN1	2.308562876	1.57E-45
ENSG00000260851	2.308995571	2.51E-07
LINC00222	2.313684897	7.26E-05
ENSG00000267990	2.314678047	2.88E-13

ENSG00000267895	2.314999812	2.46E-11
ENSG00000290003	2.319337493	1.65E-12
RN7SL517P	2.320082014	1.09E-07
ENSG00000279432	2.320402939	4.59E-22
SIRPB3P	2.323747926	3.99E-10
GPR82	2.324459566	2.92E-08
ENSG00000259453	2.324940198	1.12E-07
WNK2	2.326085255	4.95E-10
ENSG00000275902	2.32801723	2.18E-11
LLPH-DT	2.328472767	1.10E-04
TMEM154	2.330838136	8.14E-113
MALAT1	2.334619606	1.71E-49
CKLF-CMTM1	2.336591033	1.74E-05
ENSG00000254648	2.340784582	2.88E-04
ENSG00000280353	2.341957864	4.66E-09
ENSG00000260672	2.344038579	4.43E-09
ENSG00000256591	2.34560564	3.40E-43
LINC00173	2.347705127	3.05E-32
SLC6A8	2.349700627	3.29E-06
MIR6813	2.350076345	4.36E-08
ENSG00000286044	2.350294802	2.09E-05
VNN2	2.351349021	1.80E-28
ENSG00000272914	2.353964428	2.72E-13
CLEC18A	2.354771222	1.32E-11
ENSG00000278863	2.355763258	8.94E-05
IFI6	2.356270466	1.21E-07
ENSG00000290074	2.357064027	2.63E-08
ENSG00000236352	2.35772606	6.72E-06
ENSG00000205740	2.361266069	1.48E-08
SNORC	2.363963096	1.86E-27
ENSG00000277744	2.365799982	1.90E-15
ENSG00000280069	2.367431463	5.35E-13
FRY-AS1	2.369343394	1.80E-07
ENSG00000254094	2.370079719	8.76E-08
BTG2-DT	2.372354196	1.25E-12
ENSG00000289031	2.372612021	8.64E-04
ENSG00000290084	2.37572514	5.16E-07
ESPN	2.377216758	1.21E-08
ENSG00000289044	2.377456809	7.99E-09
ASAP1-IT2	2.377567228	3.44E-12
CAPNS2	2.379576525	8.29E-05
FPR1	2.379877728	3.08E-41
HYMAI	2.381547529	1.87E-04
ENSG00000267546	2.381583809	1.09E-09
RN7SKP78	2.383399869	1.19E-08
ENSG00000288896	2.383796266	7.18E-10
ENSG00000279573	2.384412857	5.27E-09

KLF1	2.385007448	1.58E-04
TLR6	2.38969005	4.07E-52
NOTCH2NLA	2.39006425	1.45E-14
DISC1-IT1	2.391205362	4.25E-05
SLC19A1	2.392007643	3.91E-40
LINC02908	2.392578406	2.81E-21
ENSG00000268119	2.393765017	7.30E-04
ENSG00000267457	2.393843455	1.97E-07
IL5RA	2.393931795	3.89E-15
ENSG00000279801	2.397039676	2.00E-16
ENSG00000271855	2.399853228	2.48E-15
RPS3AP38	2.40388097	6.39E-07
MINDY1	2.404670154	1.90E-28
ENSG00000265478	2.406725767	5.57E-05
ENSG00000286327	2.406971029	4.05E-09
FCGR2A	2.409121241	8.94E-40
ENSG00000288988	2.40914333	1.15E-08
ENSG00000237788	2.411388568	2.83E-10
ENSG00000254288	2.411659238	1.09E-20
ENSG00000279294	2.411836317	1.40E-06
SMPDL3B	2.41231434	3.71E-14
SNORA30	2.412969674	1.71E-05
C5orf67	2.415069905	1.28E-06
SLC22A4	2.41617272	3.62E-26
ENSG00000213279	2.417405475	9.61E-24
ENSG00000278949	2.418889334	4.46E-19
NAIPP4	2.419281479	4.90E-04
ACTBP4	2.419802456	1.18E-05
PHF5AP1	2.42410519	4.84E-04
ENSG00000287236	2.424220078	1.53E-04
SPCS2P4	2.425754412	2.30E-08
ENSG00000233799	2.426179897	8.48E-09
ENSG00000255186	2.426681029	1.14E-05
NOTCH2NLB	2.427425988	1.84E-24
RN7SL146P	2.429112091	7.67E-06
CREB5	2.429568499	2.08E-38
ENSG00000260911	2.430351616	5.63E-33
ENSG00000279608	2.431231031	1.94E-29
SLC23A3	2.432290191	7.54E-13
ENSG00000285646	2.432702603	6.74E-04
KPNB1-DT	2.434237784	2.40E-31
THBD	2.436294956	1.50E-15
ENSG00000279544	2.43731345	7.31E-12
PAQR6	2.437749589	1.18E-21
MIR142HG	2.437951592	7.43E-39
ENSG00000290100	2.438002362	3.82E-04
TMEM11-DT	2.43940661	9.61E-15

ENSG00000271554	2.440610801	9.48E-04
ORM1	2.443456055	2.28E-05
ENSG00000256325	2.44464572	2.82E-04
ENSG00000238035	2.446432165	5.43E-16
ENSG00000279500	2.44733969	5.72E-17
ENSG00000279281	2.447905953	1.90E-09
ENSG00000262248	2.448425674	6.95E-04
DLEU2	2.450007084	2.37E-40
TMEM45B	2.450495927	5.77E-20
CYSTM1	2.451140061	2.54E-38
ENSG00000266934	2.455206228	1.74E-09
DNASE1L2	2.458639273	8.00E-17
TNFRSF9	2.460578207	9.87E-35
ENSG00000289472	2.464357025	3.38E-05
MADCAM1	2.466371035	4.57E-26
GK	2.468575688	6.46E-35
ENSG00000271789	2.468783706	4.26E-10
ENSG00000239415	2.470474972	8.47E-13
LINC02207	2.472118666	2.66E-15
RNU6-125P	2.472320184	2.25E-06
ENSG00000289296	2.47637115	5.25E-06
GRAMD1C	2.477560917	2.38E-17
ENSG00000278829	2.481464914	5.42E-16
CTDNEP1P1	2.482425022	6.61E-11
ACSL1	2.483165902	1.23E-12
HOTAIRM1	2.484130594	3.83E-24
ENSG00000279283	2.489325613	1.39E-21
BEAN1-AS1	2.49278401	4.34E-09
TGFA	2.495511782	3.25E-30
SMYD3-IT1	2.497341623	7.55E-05
ENSG00000276744	2.500468839	1.22E-06
ENSG00000275569	2.500890579	5.92E-05
CHD1-DT	2.501535864	1.27E-48
ENSG00000232748	2.502257633	6.88E-09
ENSG00000280474	2.503772545	2.52E-06
MAP1LC3A	2.503956432	2.04E-30
HTR3B	2.504087031	4.09E-04
ENSG00000265800	2.504995369	2.34E-07
SEMA3B	2.507858623	1.12E-20
ENSG00000272630	2.510932505	2.57E-30
ENSG00000272941	2.511228796	5.25E-51
ENSG00000266385	2.515293092	3.57E-07
CSF2RB	2.518763065	2.14E-57
RNA5SP242	2.52195839	4.08E-08
CACNB4	2.521990581	1.67E-16
ENSG00000286488	2.524453507	1.96E-41
MIR1249	2.525465785	3.52E-05

CR1L	2.529514294	1.23E-07
SPACDR	2.52952892	3.37E-23
SEPTIN4	2.52996175	1.51E-04
RN7SL749P	2.533211103	9.44E-05
ENSG00000272810	2.534328858	3.71E-06
ENSG00000282742	2.534502631	1.51E-04
ENSG00000264772	2.53628336	1.26E-04
ENSG00000267474	2.539311963	2.25E-18
BNIPL	2.539969092	5.90E-22
ENSG00000288772	2.541273146	6.79E-05
SCAT8	2.54159852	2.86E-22
SND1-IT1	2.542039649	3.16E-09
TECPR2	2.543368955	4.13E-59
SCARNA20	2.544330755	7.25E-04
REPS2	2.54465868	5.17E-59
ENSG00000273763	2.545425309	7.26E-10
TRAJ26	2.546306563	2.19E-07
H2AZ2P1	2.546711104	9.45E-13
ENSG00000286872	2.546939577	1.47E-07
ENSG00000276593	2.549685104	6.06E-04
RNU6-828P	2.552664511	3.30E-04
CLEC18B	2.557181007	2.74E-12
RNA5SP195	2.558385153	4.14E-05
CD177	2.559786862	1.71E-05
ENSG00000289618	2.560027632	1.88E-07
ENSG00000276957	2.560965616	3.48E-05
ENSG00000274525	2.561014956	1.93E-11
ENSG00000256448	2.561459496	2.10E-28
IRAG1-AS1	2.562122358	1.61E-09
ENSG00000275481	2.562789247	5.54E-10
ENSG00000259797	2.563060524	3.95E-07
GPR141BP	2.563311957	1.91E-05
ENSG00000276649	2.566121063	3.37E-32
PRPF19-DT	2.567872777	7.42E-05
ENSG00000279048	2.568843917	1.07E-11
ENSG00000265791	2.570895566	1.29E-09
ENSG00000264739	2.57296606	2.02E-08
ENSG00000287737	2.574821404	1.25E-24
ENSG00000206976	2.576600927	7.45E-04
LINC01127	2.577110835	5.03E-32
STX3	2.577240523	2.30E-63
ENSG00000287917	2.577791955	5.81E-22
HSPE1P18	2.57859976	1.02E-20
RXRB	2.578613552	6.11E-04
ENSG00000272689	2.578941183	7.47E-04
ALPK1	2.579509986	3.02E-55
ENSG00000258620	2.580150512	7.25E-04

RPL17P48	2.585720587	5.97E-06
GGT5	2.586714423	4.51E-12
ENSG00000223969	2.59138865	2.38E-22
ENSG00000286076	2.59472626	1.94E-04
ENSG00000255306	2.596125446	1.54E-04
ENSG00000288757	2.598033091	1.96E-27
DAPK1-IT1	2.602718874	7.90E-04
XBP1P1	2.602729806	4.67E-06
ENSG00000280214	2.603424984	2.41E-26
ADAMTS7P4	2.604926804	7.65E-06
IFITM3	2.605481184	1.53E-11
ENSG00000289327	2.605866603	2.34E-04
TMEM140	2.608286727	1.20E-36
LPAR2	2.6087072	3.45E-49
ENSG00000288593	2.608721067	5.67E-05
ENSG00000275468	2.609057804	2.62E-11
ENSG00000269514	2.609941658	4.24E-24
ENSG00000269949	2.610030162	6.55E-06
ENSG00000279555	2.610575147	1.21E-23
CFAP54	2.610775896	8.06E-09
HNRNPA1P52	2.611047392	1.29E-10
ENSG00000290385	2.611157974	6.97E-27
ENSG00000279433	2.612354313	3.02E-17
C1QTNF7-AS1	2.612448363	9.36E-04
KRT18P4	2.614351066	3.99E-11
ENSG00000287644	2.615223617	9.89E-17
ZDHHC18	2.615817371	5.17E-59
ENSG00000271344	2.616928384	2.84E-11
ENSG00000288821	2.617287063	3.06E-04
HAL	2.618535328	4.02E-63
MIR3945HG	2.620872446	1.51E-07
EPHB1	2.62107026	5.24E-34
ENSG00000280163	2.622356996	2.83E-18
ENSG00000261692	2.623579027	4.87E-05
ENSG00000280392	2.625707508	1.24E-09
ENSG00000256433	2.627157838	1.06E-16
ENSG00000289507	2.627323697	2.34E-18
LNCATV	2.629014266	2.66E-38
APOBEC3A	2.629603329	7.90E-15
ENSG00000275401	2.629801306	8.21E-05
DAP3P1	2.632880262	1.74E-04
ENSG00000288827	2.638157129	1.47E-39
LINC02983	2.639552271	3.38E-08
ATG16L2	2.639732006	2.88E-57
MBOAT2	2.641281059	3.06E-42
MBOAT7	2.642531596	1.18E-11
ENSG00000280381	2.64296118	2.44E-11

ENSG00000273272	2.648165095	3.32E-15
MAP1LC3B2	2.648264033	2.91E-18
ENSG00000290038	2.649788495	2.29E-04
MXD1	2.649925559	2.11E-27
ENSG00000286531	2.652048953	1.79E-04
IL1R1	2.65410093	4.10E-28
ENSG00000279106	2.655015822	3.51E-18
SEMG1	2.656006198	5.15E-04
ENSG00000261804	2.656180342	7.71E-22
ENSG00000280121	2.657015962	3.34E-11
HHATL	2.657847389	2.83E-04
NCF1C	2.65885882	2.90E-46
SLC5A9	2.659719353	4.04E-14
ENSG00000266783	2.662013364	1.75E-09
GAPDHP43	2.663949049	1.74E-07
ENSG00000271553	2.664223013	1.22E-22
CD274	2.668820493	6.19E-09
ENSG00000283907	2.670396167	1.28E-11
RNU6-407P	2.675569456	5.20E-05
GPR52	2.679198339	3.18E-09
ENSG00000287707	2.679442703	3.31E-05
ENSG00000289038	2.684128371	1.28E-05
LINC02809	2.688187908	5.38E-07
MKRN5P	2.689531359	8.99E-04
DNAAF11	2.690771948	4.03E-19
ENSG00000261916	2.693337387	1.41E-05
ENSG00000257298	2.693495844	5.78E-04
CHRNA10	2.69881975	3.44E-52
ENSG00000263120	2.703768293	1.11E-06
ENSG00000274693	2.704165979	6.27E-04
ENSG00000279819	2.70435203	4.44E-08
CCDC13-AS2	2.70692826	3.13E-19
ENSG00000288881	2.707301033	6.62E-06
ENSG00000260425	2.708881875	7.09E-10
MIR1537	2.709581841	4.96E-04
ENSG00000260070	2.714077089	2.67E-06
NTNG2	2.715379114	1.48E-36
ENSG00000277463	2.716456362	5.42E-06
C13orf46	2.717590039	1.12E-07
ENSG00000285444	2.719608557	4.02E-15
GCA	2.722278866	7.32E-52
ENSG00000262514	2.725190096	1.39E-15
ENSG00000289164	2.725995055	5.00E-08
ENSG00000291221	2.727644618	9.89E-07
RNU2-6P	2.728565852	7.36E-07
NAV3	2.731169932	3.41E-04
INHBB	2.732729638	1.50E-05

CDRT4	2.732913244	1.89E-04
ENSG00000273139	2.733075112	6.24E-08
ENSG00000279900	2.733169751	2.35E-07
RERE-AS1	2.735071977	2.46E-33
PLEK2	2.737169671	1.49E-12
ENSG00000288704	2.737949046	3.86E-08
ENSG00000267174	2.739780161	6.33E-33
LIMK2	2.741404378	3.32E-25
RSPH14	2.741783171	1.97E-14
TEDC2-AS1	2.743133285	6.77E-04
ALDH1A2	2.744156277	6.60E-12
ENSG00000289023	2.744170226	2.94E-07
DNAH7	2.745893544	6.17E-04
LINC00656	2.749432473	4.51E-05
BASP1	2.750282887	1.47E-30
ENSG00000287892	2.750330506	1.68E-06
RPS26P42	2.751029121	1.80E-04
ENSG00000288848	2.754271829	4.08E-06
DOCK4	2.754442322	5.89E-19
ENSG00000261056	2.756252315	8.44E-06
RNU6-920P	2.760489855	5.18E-09
H2BC4	2.760622791	2.04E-30
H4C5	2.763400416	1.36E-15
LST1	2.763578975	2.05E-13
ENSG00000279811	2.764811857	1.65E-18
ITGB4	2.765101025	1.82E-16
MSANTD3P1	2.765166916	4.49E-04
DYSF	2.769268827	2.24E-32
C10orf105	2.769926046	1.10E-50
ENSG00000289371	2.770114341	1.83E-04
RPL17P10	2.771060843	5.24E-10
CACNG8	2.773392943	2.48E-20
ENSG00000289045	2.775342158	3.37E-17
AQP9	2.777106609	5.99E-21
PIRAT1	2.778793445	2.01E-16
ENSG00000268987	2.779083786	1.63E-04
REM2	2.779226499	1.04E-35
SNX30-DT	2.781367364	3.62E-06
ENSG00000290318	2.781896477	4.51E-05
ENSG00000278075	2.782086094	6.06E-04
ENSG00000285091	2.782390626	1.54E-04
ENSG00000277728	2.786527855	3.28E-15
ENSG00000262714	2.786821985	1.16E-34
TREML2	2.786854412	3.16E-54
OR8R1P	2.78785864	9.98E-06
ENSG00000272669	2.788616339	1.24E-33
ACOXL	2.791644321	5.91E-08

ENSG00000272010	2.792371585	2.20E-07
SHISA4	2.800295494	9.19E-22
FCGR1BP	2.800856001	2.60E-13
APOL4	2.803433079	4.43E-06
ENSG00000283341	2.808856337	6.98E-34
HECW2	2.809085564	1.01E-17
HSPA8P14	2.809527089	9.12E-04
NCF1	2.815822123	1.95E-69
ENSG00000269194	2.816185151	2.40E-19
ENSG00000227200	2.818299087	3.73E-08
NCF1B	2.818614265	6.32E-48
ENSG00000274765	2.819232742	5.58E-08
MPHOSPH10P1	2.819739332	1.38E-51
NFE2	2.820763188	2.36E-30
ENSG00000271752	2.824830156	7.04E-05
ENSG00000287387	2.829756107	6.06E-16
ENSG00000285851	2.831266524	4.67E-23
ENSG00000250189	2.831590544	1.38E-07
RNU6-942P	2.834823022	6.64E-06
OXER1	2.835682804	3.46E-87
ENSG00000286342	2.836402398	1.82E-05
ABTB1	2.837923949	3.08E-99
RPL12P27	2.8382313	1.90E-14
MIR3677	2.841447728	3.32E-06
ENSG00000260757	2.842635627	3.39E-05
GCM1	2.842760405	4.58E-14
RNF24	2.843989957	3.72E-61
ENSG00000269560	2.848498652	1.54E-12
LINC01814	2.849296314	6.39E-23
IFITM3P1	2.852587609	4.00E-06
MIR320E	2.855058901	4.38E-16
ENSG00000272948	2.857449022	1.54E-07
LINC01342	2.860465808	3.07E-07
MIR3609	2.861465731	3.80E-11
ENSG00000286782	2.861920633	1.92E-20
MNDA	2.862528713	9.85E-51
ENSG00000275278	2.867997362	4.47E-04
ENSG00000259376	2.868454763	2.27E-08
RCN3	2.870381577	5.52E-50
ADORA3	2.874765323	1.33E-15
NFE2L1-DT	2.874906235	9.36E-17
LINC02363	2.875217804	2.04E-19
MIR4518	2.875718078	9.66E-09
HBM	2.882049448	4.93E-05
B3GNT8	2.884017432	1.06E-49
FAM157C	2.884334762	3.92E-27
ENSG00000226576	2.884914887	6.53E-04

LINC01506	2.886171435	2.38E-29
ENSG00000224789	2.886552679	2.14E-04
PRRG4	2.888428316	4.04E-27
TMEM88	2.888706351	1.12E-23
ENSG00000269385	2.890473693	8.06E-05
ENSG00000280027	2.891744278	2.37E-25
ENSG00000273797	2.892200171	3.07E-10
ENSG00000225750	2.893900756	2.62E-04
LINC00671	2.89602344	2.51E-19
ENSG00000290035	2.896900636	5.59E-05
ENSG00000290108	2.899406438	1.38E-08
ENSG00000225450	2.900048958	1.51E-24
DHX34	2.900464521	4.65E-85
ENSG00000266965	2.901838272	2.82E-07
VNN3P	2.90247277	1.97E-30
MIR29B2CHG	2.903951611	2.18E-57
ENSG00000259755	2.905601208	6.20E-07
ENSG00000288965	2.908221666	2.57E-08
H3-3A-DT	2.90869786	2.83E-16
ENSG00000255801	2.910905867	1.79E-13
ENSG00000225889	2.912565598	3.08E-54
ENSG00000287255	2.91259437	1.98E-18
CTSE	2.917430278	2.71E-08
SCARNA7	2.919897026	2.24E-19
PDLIM7	2.92140945	1.06E-54
CXCL6	2.922109007	1.84E-06
ENSG00000235683	2.922693965	2.54E-04
ENSG00000285816	2.92340624	1.53E-18
ENSG00000289281	2.923766513	1.48E-05
CNTNAP3C	2.923964387	5.49E-04
ENSG00000286533	2.925931082	3.78E-04
LINC02080	2.925988751	2.61E-05
NSMCE1-DT	2.926048566	2.74E-14
SLC4A9	2.929521081	7.39E-04
PHB1P19	2.933941842	2.03E-18
ENSG00000289584	2.934829723	1.94E-14
ENSG00000290022	2.938857009	4.00E-04
ARHGEF2-AS2	2.941261755	2.55E-46
ENSG00000267749	2.945039943	1.29E-15
P2RY13	2.945422768	6.33E-57
LINC01001	2.946517795	1.39E-30
ENSG00000279977	2.947962303	4.74E-15
FCGR1CP	2.950743628	1.26E-07
GOLGA5P1	2.95527319	1.45E-18
DRC1	2.956462412	2.03E-08
PGAM1P11	2.957283085	1.80E-05
ENSG00000267283	2.959220113	1.41E-35

H3C6	2.95934302	1.03E-17
TSPAN5-DT	2.959971053	2.42E-08
ENSG00000228886	2.965264167	8.60E-09
ENSG00000280537	2.968540177	2.26E-13
ENSG00000240669	2.972975451	5.60E-07
ENSG00000288748	2.974139081	3.58E-23
FPR2	2.978879463	2.49E-46
ENSG00000289067	2.980066629	1.79E-10
CLEC18C	2.981082095	4.37E-04
ENSG00000270019	2.982155327	1.31E-10
CEP295NL	2.98395567	9.05E-20
ARAP3	2.985484037	6.08E-58
MIRLET7BHG	2.985563797	3.58E-49
ENSG00000267940	2.987288955	1.96E-27
STBD1	2.988460214	6.08E-04
ZNF252P-AS1	2.989552399	3.61E-16
RPS2P44	2.98977732	2.34E-10
CFAP276	2.997559358	5.81E-05
MAB21L3	2.999491031	1.86E-09
SCARNA3	2.999708452	2.70E-07
ANXA3	3.005084572	2.00E-15
CA15P1	3.0072348	1.32E-30
MIR194-2HG	3.007713492	2.46E-14
MTND1P11	3.009812722	1.10E-08
ENSG00000280157	3.010578394	3.68E-12
RPSAP36	3.011202615	5.57E-07
ENSG00000288860	3.012868733	9.73E-08
ENSG00000253399	3.01592292	9.33E-10
RN7SL587P	3.016288421	5.56E-08
MIR3679	3.031187857	4.49E-04
FRAT2	3.036075145	9.93E-109
ENSG00000272906	3.037845739	7.16E-29
MIR661	3.038705211	6.65E-05
ENSG00000287200	3.039852421	8.54E-34
LSMEM1	3.041457453	2.10E-20
RN7SKP292	3.043036013	1.40E-06
SLC45A4	3.043600369	3.58E-78
ENSG00000290585	3.044305479	9.99E-21
ENSG00000289196	3.048515495	1.16E-11
HBQ1	3.054601072	2.37E-09
ENSG00000253475	3.058370263	3.95E-07
CDC42-IT1	3.059432263	6.60E-18
ISL2	3.060111907	2.66E-19
FOXP1-DT	3.06689379	2.82E-26
ERBIN-DT	3.068541454	1.77E-08
ENSG00000278972	3.069065579	8.39E-07
LRRC4	3.070015003	5.09E-36

H1-5	3.070808684	3.52E-04
FHDC1	3.07352024	2.89E-20
DGAT2	3.074656713	2.57E-42
ENSG00000279434	3.076988223	2.43E-09
ENSG00000273302	3.081916125	5.32E-15
HBG2	3.083053658	7.59E-04
IRAG1	3.083140589	4.58E-53
ENSG00000273133	3.083440916	2.95E-31
ENSG00000262979	3.089109417	1.80E-14
ENSG00000289017	3.091132738	2.17E-24
ENSG00000260859	3.091815251	1.19E-05
LINC02967	3.092444418	4.02E-06
ENSG00000290062	3.09635458	8.05E-22
IGSF6	3.096424715	6.95E-56
RN7SL127P	3.09726437	7.08E-05
LINC02649	3.097644583	3.99E-58
DRAIC	3.097871447	5.76E-27
SLPI	3.101977229	2.04E-15
ENSG00000267412	3.103536177	6.74E-09
EXOC3L1	3.106675483	3.40E-11
ENSG00000288863	3.106966264	8.49E-06
RN7SL3	3.107571523	5.72E-08
NCF4	3.109926603	1.81E-74
BEST1	3.110661336	4.94E-71
DHRS9	3.110839888	3.26E-14
ENSG00000263786	3.112155579	3.07E-15
ENSG00000274322	3.112747429	1.18E-09
ENSG00000267512	3.114169491	2.35E-08
ENSG00000279320	3.118361227	3.13E-20
DUSP13	3.118655092	3.20E-09
ENSG00000269399	3.11984978	2.67E-30
NDUFB8P2	3.121981513	6.28E-06
TAS2R3	3.124027639	2.81E-06
CCNJL	3.126789909	1.52E-29
ENSG00000286288	3.128361408	1.75E-45
RN7SL45P	3.131802682	4.72E-07
ENSG00000273076	3.134613154	1.35E-10
ENSG00000279527	3.13473157	2.30E-04
MIR3613	3.135189303	7.65E-09
ENSG00000288745	3.135962121	1.98E-10
ELAPOR1	3.13849979	2.69E-51
LINC03062	3.138925271	3.72E-06
SZT2-AS1	3.142183645	1.64E-05
SMIM1	3.148483929	4.99E-13
ENSG00000282416	3.149540079	3.65E-06
RNU6-890P	3.15163193	2.67E-09
SIGLEC5	3.1516897	6.67E-41

LINC01765	3.155778131	1.21E-09
SPACA6	3.156402098	1.06E-59
ENSG00000270190	3.157260683	8.31E-05
IFITM2	3.164845933	3.35E-61
ENSG00000241860	3.165696064	2.30E-33
KCTD9P1	3.171356221	1.07E-08
CCR3	3.175892778	5.54E-41
GSEC	3.176173695	4.05E-54
TNFSF14	3.17962062	3.58E-89
ENSG00000289331	3.181288029	8.79E-25
LSMEM2	3.182744179	9.03E-08
PRDM5	3.185262487	1.09E-22
MARK2P18	3.190663984	3.51E-06
ENSG00000275557	3.191444473	9.74E-11
ANKRD44-IT1	3.192701936	4.28E-33
ENSG00000254826	3.192890333	1.57E-10
RSAD2	3.193646519	5.30E-08
H4C6	3.194199561	1.12E-04
LINC03078	3.195533909	1.96E-07
ENSG00000250069	3.197186972	1.55E-12
IL34	3.199175849	8.86E-10
RN7SL698P	3.206300979	5.58E-09
ENSG00000260417	3.20681387	8.79E-26
FAM83E	3.20724405	6.75E-09
EYS	3.209553326	5.25E-10
ENSG00000276494	3.213515227	9.73E-05
BEND7	3.214015396	6.88E-16
ENSG00000254477	3.215508196	1.51E-21
ENSG00000266498	3.215647533	2.35E-09
C5AR2	3.219008683	1.20E-91
TIMMDC1-DT	3.221818368	1.54E-04
OTULIN-DT	3.223229222	3.50E-11
LRRK2-DT	3.228991606	3.03E-42
ENSG00000267082	3.229457413	1.02E-05
ENSG00000289382	3.233636109	1.38E-17
OR52K1	3.233879968	1.32E-19
ATP6V0CP4	3.240549809	2.01E-06
METTL21AP1	3.243181133	1.34E-05
MIR7845	3.243512705	2.37E-04
ENSG00000276105	3.244607326	9.72E-06
SCARNA21	3.246911447	3.42E-26
TEK	3.249657392	2.28E-05
ENSG00000261218	3.251250037	3.99E-75
ENSG00000225195	3.256267764	1.30E-19
MTCYBP23	3.25732488	6.47E-07
MTND6P5	3.25832967	6.24E-08
LINC02362	3.259504111	1.38E-29

LINC01303	3.266600028	1.09E-11
H1-4	3.267248468	3.07E-25
HEY1	3.268879059	4.16E-08
ETV7	3.269280447	4.17E-11
C3orf86P	3.269348441	8.97E-53
ENSG00000273064	3.27368383	3.33E-17
ST20	3.276657898	3.86E-50
ACKR1	3.283934374	4.67E-05
ANKRD22	3.286630507	9.87E-09
LINC01762	3.293246996	4.42E-23
ENSG00000274737	3.293469272	2.22E-16
MIR6842	3.295510471	2.11E-05
RN7SKP16	3.295630356	1.92E-15
ENSG00000266111	3.299883026	2.34E-09
ENSG00000289353	3.303036585	1.59E-27
ENSG00000264546	3.304822354	5.08E-12
LINC02217	3.306653931	2.30E-07
ENSG00000250574	3.307048203	2.58E-13
ENSG00000241772	3.309627863	1.22E-07
RNF182	3.314032554	6.43E-04
MIR3605	3.316033108	4.54E-12
RPS3P2	3.316852642	1.08E-08
FDX2	3.322261073	6.49E-07
ENSG00000187904	3.325885648	1.39E-11
RN7SL57P	3.328536031	7.53E-05
INKA2	3.335923086	5.42E-97
CEACAM1	3.345755105	2.74E-19
TNFAIP6	3.346202376	2.58E-09
ENSG00000253986	3.346670313	4.04E-05
ENSG00000273973	3.347479833	1.70E-07
POU5F1P5	3.352373954	1.62E-06
ENSG00000285730	3.355423059	1.91E-17
CSF3R	3.36622379	1.92E-63
MIR604	3.36673679	2.23E-05
ENSG00000289379	3.367721081	1.02E-05
ENSG00000275485	3.377568157	7.13E-08
RN7SL220P	3.379026191	5.52E-08
ENSG00000279199	3.379354767	7.67E-10
ENSG00000279202	3.380395286	4.43E-18
IFIT1	3.380953424	3.53E-11
ENSG00000232334	3.388115105	2.74E-22
MIR5194	3.401254818	6.52E-16
FAM157D	3.401546365	3.24E-26
ENSG00000277825	3.40195708	2.95E-06
ENSG00000257169	3.406032556	5.49E-06
KCNJ2	3.406495148	1.07E-12
LYVE1	3.413639889	3.35E-14

SEPTIN5	3.417554364	4.34E-15
ENSG00000279447	3.421471821	2.09E-24
BAZ2B-AS1	3.424358041	5.56E-46
CFAP58-DT	3.426741842	3.20E-18
ENSG00000273691	3.427504453	9.39E-29
MTND5P2	3.429440539	1.05E-14
KCNS1	3.432085704	4.68E-06
ENSG00000289534	3.436425886	8.53E-10
ENSG00000259600	3.442462528	2.41E-11
ENSG00000270210	3.443012682	1.16E-44
RNA5SP307	3.445541628	2.66E-08
SHISA7	3.446851304	4.03E-07
CEP19	3.447236526	4.41E-35
BACH1-IT3	3.449701084	1.09E-05
BLZF2P	3.456095259	1.54E-17
ENSG00000276166	3.456848567	8.97E-08
IFIT2	3.457020299	1.23E-21
HCAR3	3.457943113	2.90E-19
ENSG00000258632	3.458039288	1.93E-05
XPC-AS1	3.46436162	1.78E-79
ENSG00000265728	3.46516694	6.05E-06
KLC3	3.466846653	3.57E-05
FOXP1-IT1	3.468499813	1.40E-19
ENSG00000279598	3.469129075	5.32E-18
LINC00683	3.470053479	3.61E-12
CDH2	3.470903541	4.50E-10
C4BPA	3.471224984	8.52E-05
ENSG00000274717	3.47279442	7.67E-17
ENSG00000232528	3.472949483	2.50E-28
ENSG00000243273	3.476280183	3.57E-20
ENSG00000273381	3.476566608	1.80E-06
ENSG00000288853	3.477176446	2.74E-05
MIR223HG	3.47748883	2.14E-57
RNU7-181P	3.481620597	5.25E-15
TRPM6	3.482772735	2.79E-34
RN7SL521P	3.487037663	1.71E-08
STAM-DT	3.490693868	1.77E-08
ENSG00000287064	3.497225597	2.14E-08
SUMO1P1	3.499408584	3.42E-11
ENSG00000286830	3.500180426	4.38E-09
KRT18P31	3.500949158	9.51E-16
LUNAR1	3.501458649	1.11E-20
RN7SL648P	3.501684841	2.90E-17
ENSG00000284602	3.502444422	1.58E-17
ENSG00000269981	3.505300989	2.92E-21
SLC25A37	3.506742186	4.33E-35
ADGRE3	3.50684039	1.36E-85

LINC02772	3.50984296	1.65E-05
DMRTC2	3.513517313	1.33E-07
NSUN7	3.515134539	2.36E-30
ENSG00000292299	3.516458588	3.14E-09
ENSG00000283384	3.518499745	6.72E-11
ENSG00000285572	3.522239809	1.29E-13
ENSG00000239480	3.525411109	8.78E-11
LINC01002	3.527875012	1.07E-34
DPEP3	3.530743205	6.15E-52
FLJ42393	3.544678189	3.04E-16
CBX3P10	3.546179366	5.15E-10
RPS16P5	3.547298395	1.85E-09
RPS15AP29	3.549212528	1.64E-12
MTND5P32	3.549665546	3.32E-09
RPS29P14	3.550880989	7.79E-06
CYP4F12	3.552936645	1.72E-35
ENSG00000271327	3.554638273	8.47E-22
RNU1-134P	3.558089098	1.66E-13
ENSG00000286236	3.558453998	7.07E-09
OOSP3	3.559102032	1.33E-06
RASA2-IT1	3.563007677	1.48E-10
ENSG00000274695	3.563471346	3.34E-06
MTND3P12	3.565969315	9.21E-10
POU5F2	3.572607438	2.40E-28
ENSG00000237249	3.572807686	6.60E-06
RNU4ATAC11P	3.573443581	1.65E-06
RNU6-176P	3.573746383	2.59E-11
RNU6-761P	3.574401538	3.81E-16
RN7SL239P	3.581679526	3.68E-14
TPRKBP2	3.584999271	1.54E-16
LINC00896	3.585078115	8.49E-14
ENSG00000234208	3.585894607	5.00E-09
PEAK3	3.588294977	6.58E-23
ENSG00000286314	3.588466817	7.54E-22
NEAT1	3.589441287	1.66E-60
ENSG00000250575	3.593667177	2.86E-11
PROK2	3.597588231	8.52E-20
DAPK2	3.598898131	1.75E-72
ENSG00000226862	3.60394335	3.02E-31
MIR4477B	3.604698568	2.63E-31
ENSG00000230615	3.610693595	3.32E-11
ENSG00000290077	3.612162114	3.21E-06
ENSG00000231970	3.614986932	2.46E-05
SERPING1	3.61501454	4.55E-10
ENSG00000273451	3.617927316	2.13E-09
IL1R2	3.619555527	9.11E-22
ENSG00000260361	3.622442054	5.71E-56

ENSG00000239906	3.622906705	5.73E-10
ENSG00000241886	3.62470269	2.39E-19
RC3H1-IT1	3.625765253	6.78E-17
ENSG00000268903	3.634181329	5.13E-35
ENSG00000230454	3.634334286	2.09E-27
ENSG00000279814	3.636405837	7.14E-10
ENSG00000288836	3.642525351	8.54E-11
HCG27	3.645825458	6.69E-07
AGAP7P	3.651200242	1.55E-15
ENSG00000288082	3.65489891	8.93E-11
CFAP58	3.658258401	4.76E-18
MAP3K15	3.658720605	8.35E-13
AVIL	3.65928117	3.18E-35
TIGD3	3.662613643	2.95E-68
LRG1	3.663818348	8.74E-23
STEAP4	3.66386007	3.11E-66
INSC	3.665036154	1.80E-13
ENSG00000200075	3.666240094	3.49E-06
ENSG00000268051	3.670246427	3.10E-07
ENSG00000289923	3.670955423	2.58E-11
ENSG00000238260	3.674559871	3.35E-59
MIR4285	3.675248507	1.72E-06
APOBEC3B	3.675501762	1.51E-07
ENSG00000273597	3.676906241	1.69E-06
ENSG00000255328	3.682457386	3.33E-32
RN7SL5P	3.683296554	2.31E-11
ENSG00000227045	3.68330817	1.30E-06
TMEM252-DT	3.687543928	1.06E-08
TVP23CP1	3.694342475	7.22E-08
ENSG00000289434	3.69779632	7.07E-08
MMP9	3.699723245	9.11E-20
ENSG00000272953	3.699750021	4.99E-32
ENSG00000274427	3.710088608	2.47E-12
ENSG00000272219	3.716538406	7.72E-20
MAK	3.729047011	4.06E-31
OLAH	3.732956609	1.70E-06
E2F3-IT1	3.734149948	1.79E-09
ROPN1L	3.737037989	5.10E-54
AATK	3.738897529	9.21E-66
ENSG00000270072	3.743095304	5.72E-13
ENSG00000218596	3.746794568	8.21E-11
IGLV3-27	3.749651278	5.19E-05
ENSG00000289129	3.751188396	1.31E-06
ARF1P2	3.755614211	6.39E-10
ENSG00000289457	3.757191132	2.42E-12
ENSG00000270972	3.760669436	1.18E-28
HBA1	3.762104589	1.74E-07

ENSG00000289039	3.763059124	4.59E-15
ST6GALNAC2	3.771745629	2.78E-66
ENSG00000235008	3.772864855	7.95E-06
RPL35AP2	3.774290381	1.90E-12
ENSG00000280181	3.779545172	3.17E-12
ENSG00000289528	3.779792585	7.51E-09
ENSG00000230063	3.78128669	3.56E-11
ARHGAP26-IT1	3.782820296	1.19E-12
ENSG00000290021	3.783534132	3.39E-55
RGL3	3.784776123	5.40E-44
PDCD1LG2	3.786710038	4.53E-07
MIA	3.795061379	1.93E-09
MIR3161	3.79565419	1.51E-07
ENSG00000284930	3.795956853	1.81E-46
GBP6	3.797926566	1.69E-10
ENSG00000279821	3.801294678	9.72E-29
MGAM	3.804460831	1.94E-18
PANX2	3.82172411	5.13E-47
SFRP2	3.822356573	1.95E-06
ENSG00000259682	3.824715514	6.82E-08
ENSG00000271204	3.826411304	2.14E-25
RPL21P123	3.828279676	1.12E-24
AOC4P	3.828334683	3.37E-11
ENSG00000280035	3.829372663	9.92E-39
ENSG00000286205	3.829588595	6.45E-14
ENSG00000261226	3.833888652	1.90E-36
MYL4	3.838751601	3.32E-06
RN7SL344P	3.839505621	1.89E-07
MIR326	3.839573598	3.20E-08
PPL	3.844505525	8.46E-07
ENSG00000271151	3.849143778	2.66E-37
ENSG00000279276	3.872368676	2.38E-13
SLC22A1	3.874758721	1.64E-35
ENSG00000289085	3.878584287	3.65E-13
ZDHHC19	3.879493055	5.93E-23
SOX6	3.879608306	1.38E-12
CTBP2P8	3.893040116	1.13E-10
DPRXP2	3.904416456	1.13E-07
ENSG00000279476	3.906020111	3.34E-26
AOC2	3.926140903	2.72E-49
ENSG00000238009	3.931884151	5.39E-15
PPP4R1-AS1	3.931981089	3.49E-09
ENSG00000289059	3.932097377	1.07E-11
HSPA6	3.940126514	3.52E-64
ENSG00000272037	3.952316816	1.12E-38
MTCO2P33	3.954177578	3.33E-07
ENSG00000271993	3.974037055	1.46E-20

ENSG00000249930	3.974978162	4.89E-07
ANKUB1	3.976628887	2.87E-09
ENSG00000288667	3.977595564	3.30E-07
VWCE	3.980701609	2.17E-19
RN7SL683P	3.996499007	9.65E-09
LINC02596	4.0011544	2.23E-13
MIR26A2	4.002005416	1.73E-08
GALNT14	4.005859253	1.36E-28
ENSG00000286127	4.00966822	2.61E-10
MYCBP2-AS2	4.010773546	1.19E-08
ENSG00000279884	4.011082724	2.84E-64
HBB	4.015183659	4.66E-10
MIR150	4.017067304	2.41E-11
CA4	4.033117569	3.32E-12
ENSG00000289702	4.042000214	7.29E-36
ENSG00000275236	4.042746095	1.49E-07
GK-IT1	4.0484688	1.62E-16
ENSG00000253307	4.049511407	3.81E-22
LINC00862	4.05029637	5.26E-29
MIR3174	4.057203768	9.69E-15
RN7SL288P	4.057386812	2.66E-23
H2BC18	4.057541987	8.17E-32
CDCA4P4	4.062341041	4.37E-14
OR52K2	4.064576385	5.02E-14
ENSG00000289557	4.079753428	1.06E-33
ENSG00000262636	4.082722853	1.21E-15
HCAR2	4.087080465	3.19E-24
ENSG00000279742	4.092903746	1.91E-07
MIRLET7G	4.094697055	4.30E-08
ENSG00000290937	4.095759604	3.15E-32
PTGES2-AS1	4.105368679	7.15E-13
ENSG00000279394	4.107123516	1.16E-16
ENSG00000232680	4.114406876	4.09E-09
SPINK8	4.114454373	2.89E-18
ENSG00000270426	4.120310748	3.10E-45
IFIT3	4.120393709	8.06E-20
ENSG00000237927	4.131887361	4.81E-20
H3C11	4.134474326	7.03E-08
ENSG00000280205	4.144609227	1.85E-25
ENSG00000272719	4.147138877	3.81E-10
ENSG00000227836	4.148573906	4.74E-07
MTCO3P23	4.150621291	4.29E-12
IFITM3P2	4.151825852	5.09E-26
ENSG00000225092	4.155678766	2.64E-14
CKAP2LP1	4.156712326	5.03E-11
MIR548C	4.173861352	7.03E-11
ACTBP8	4.175517134	2.40E-04

ENSG00000272506	4.179989041	1.19E-24
ENSG00000260495	4.192669238	5.35E-23
HSPA1B	4.202836302	1.18E-16
ENSG00000258922	4.218827424	2.54E-19
SLC26A8	4.221061103	1.82E-27
RNU6ATAC39P	4.230739382	1.31E-18
ENSG00000284874	4.23896743	1.66E-15
RN7SL364P	4.245211296	4.20E-20
ENSG00000267385	4.252191161	1.31E-25
LINC01270	4.252671754	5.06E-43
ENSG00000264853	4.253885196	8.54E-39
BATF2	4.266035052	2.80E-14
CMTM2	4.285305114	9.67E-47
BASP1-AS1	4.29525195	2.00E-20
PRSS41	4.296898671	8.26E-13
ENSG00000278876	4.311391007	1.88E-18
ENSG00000289310	4.313743937	2.45E-18
IDO1	4.316298915	9.99E-17
FFAR2	4.325162486	7.02E-20
RNU7-45P	4.327899161	1.81E-23
ENSG00000272682	4.345817795	1.18E-08
CFAP92	4.350475257	5.86E-46
ENSG00000285994	4.353460004	3.66E-41
ENSG00000282988	4.359515559	2.27E-31
ADAMTSL4-AS2	4.363232362	7.19E-58
ADGRG3	4.368991259	4.84E-28
SPP1	4.371746959	6.18E-14
LINC02863	4.392918766	7.08E-63
FBXL13	4.399835645	7.47E-54
ENSG00000287926	4.403640813	3.84E-45
DAAM2	4.406116167	8.37E-11
ENSG00000271259	4.420225208	2.34E-10
RNU6-1003P	4.435484179	9.82E-10
H4C4	4.442032845	5.81E-13
ACSS3	4.447828393	2.09E-46
ENSG00000266805	4.448500943	4.80E-22
KREMEN1	4.449939709	1.49E-20
RUNDC3A	4.46463256	1.18E-15
BMX	4.480051023	6.57E-17
ENSG00000289955	4.480689341	1.55E-09
RN7SL473P	4.483407747	1.63E-37
ENSG00000285667	4.487471035	7.62E-53
RN7SL4P	4.487719789	2.93E-38
MIR1250	4.490831546	1.36E-16
ENSG00000288948	4.494826194	9.59E-25
ENSG00000285933	4.498210036	5.99E-08
ENSG00000288997	4.499255415	9.69E-24

HBA2	4.500955376	7.02E-10
ENSG00000289103	4.502942511	1.74E-12
AOC3	4.505621721	4.68E-52
ENSG00000260865	4.507385487	1.39E-11
PHF24	4.507521311	9.58E-14
RN7SL1	4.526527577	2.31E-97
ENSG00000285618	4.547329832	4.93E-26
ENSG00000290091	4.553203532	3.10E-48
ENSG00000290017	4.564441025	9.34E-23
KIF19BP	4.569433712	4.17E-09
MIR4673	4.569833937	9.85E-12
ENSG00000279957	4.573787822	3.34E-22
ENSG00000259182	4.575806253	3.00E-14
ENSG00000273424	4.57729523	3.21E-36
RAB1AP1	4.596866323	1.01E-16
ENSG00000261451	4.597535527	6.35E-10
ENSG00000285693	4.600363012	4.49E-135
ENSG00000258111	4.613062463	2.33E-28
KIAA0319	4.626122868	1.50E-33
ALPL	4.628500676	5.80E-11
ENSG00000229191	4.628685142	7.77E-24
ENSG00000279176	4.63690202	1.20E-34
KAZN	4.63831561	2.33E-43
ENSG00000258337	4.641822152	2.00E-16
ARL4AP2	4.645488954	3.66E-11
ENSG00000288592	4.652913791	6.10E-12
MIR657	4.678670057	1.83E-11
BTNL3	4.683057987	5.32E-04
LINC02972	4.689342272	1.64E-14
ENSG00000233030	4.699693864	4.12E-14
CDC42EP2	4.699901071	5.81E-12
ADAMTSL4-AS1	4.703121977	6.51E-59
CBS	4.707618281	7.60E-10
MME	4.733570568	5.52E-20
RPL30P7	4.734785493	2.32E-11
ENSG00000289768	4.74207916	8.18E-15
BRWD1-IT1	4.747238444	6.51E-11
RTEL1P1	4.757728143	3.87E-32
DNAJB6P7	4.771779335	1.01E-12
ENSG00000275898	4.778363034	2.40E-13
ENSG00000252438	4.78243364	8.94E-14
ENSG00000260152	4.787316588	1.28E-12
LINC01513	4.787588659	2.39E-26
NFE4	4.812242639	1.61E-24
ENSG00000251093	4.814255256	2.81E-16
ENSG00000289541	4.816571978	1.42E-11
RMRP	4.82041092	4.21E-66

PLIN4	4.839003143	6.76E-21
RN7SL600P	4.873768535	1.76E-37
MMP25	4.883509992	8.48E-47
ENSG00000285966	4.884925432	2.37E-13
ENSG00000256020	4.888075264	2.08E-24
ENSG00000272079	4.901298127	3.48E-60
PLIN5	4.906188173	1.05E-41
LINC00639	4.912668236	1.39E-18
ENSG00000224067	4.924818785	3.43E-39
PHOSPHO1	4.93285798	2.96E-18
CHILL1	4.936260896	2.92E-52
ENSG00000188897	4.958574742	3.58E-78
ENSG00000267262	4.967161628	1.44E-09
TNFRSF10C	4.97375843	8.50E-47
ENSG00000261471	4.978951155	6.73E-73
XKR3	4.983865251	7.10E-17
PI3	4.984359236	6.42E-10
ENSG00000233214	4.989908493	1.44E-13
ENSG00000279349	4.999956859	1.56E-22
ENSG00000289576	5.001458788	1.18E-32
CNTNAP3	5.016171769	2.59E-15
ENSG00000284526	5.026043213	5.58E-61
RNU6-1176P	5.050358242	1.75E-14
ENSG00000289013	5.069293597	2.82E-27
CXCR1	5.085843403	7.32E-27
YPEL3-DT	5.096319013	1.22E-64
LINC01359	5.102896139	7.52E-55
ENSG00000233875	5.109261193	3.81E-20
NECAB2	5.12249071	9.85E-37
LINC03061	5.124307923	1.31E-12
CYP4F3	5.128659533	1.03E-40
ENSG00000289351	5.137036495	6.01E-81
ENSG00000257715	5.141029508	1.41E-31
ENSG00000289908	5.14111864	6.35E-15
ENSG00000289478	5.171320694	1.26E-20
TMEM92-AS1	5.182764074	8.84E-22
LINC02218	5.188407069	6.69E-54
RNA5-8SN4	5.202511503	1.18E-11
CXCR2	5.212893054	2.33E-34
CLDN9	5.219076327	2.93E-83
OLIG2	5.249190426	1.64E-13
ENSG00000257878	5.258240109	1.90E-33
ENSG00000275110	5.260374177	1.89E-20
CCN3	5.273040117	6.21E-68
ENSG00000287131	5.27552962	2.62E-21
SLC2A3P2	5.276059827	2.64E-26
RNU6-759P	5.282743513	5.46E-17

ENSG00000288700	5.290134662	2.92E-20
ENSG00000280248	5.307468837	9.70E-29
LUCAT1	5.327240138	3.47E-113
ENSG00000230492	5.327668948	1.26E-22
ENSG00000254789	5.34139647	4.73E-10
FAM169BP	5.379985145	7.05E-17
LINC02555	5.392742972	1.68E-16
ENSG00000284948	5.400743393	1.66E-60
TSPEAR	5.411486439	4.52E-15
KCNJ15	5.429475933	9.66E-35
FCGR3B	5.43815186	1.24E-19
LINC02289	5.472181775	1.22E-18
CHI3L1	5.47466005	1.37E-30
ENSG00000289172	5.491167225	7.04E-19
CEACAM3	5.498953715	2.51E-109
ENSG00000288794	5.509591273	1.49E-17
CORIN	5.512423791	1.14E-13
ENSG00000279996	5.617727248	1.49E-16
OR51R1P	5.627068701	7.48E-20
ENSG00000260592	5.640243585	3.60E-24
ENSG00000287201	5.650298767	2.94E-41
OTX1	5.701519652	5.17E-44
ENSG00000254325	5.71714364	6.40E-60
PRSS33	5.71919478	1.60E-24
ENSG00000268170	5.730223444	1.49E-22
CACNA1E	5.736654151	6.03E-22
MYBPH	5.819366446	3.53E-55
ENTPD2	5.821710006	1.48E-21
ENSG00000261367	5.828894941	4.63E-20
ENSG00000268240	5.85364863	1.28E-16
ENSG00000288932	5.89292488	9.43E-93
BTNL8	5.919965526	3.88E-37
ENSG00000289561	5.982867606	3.57E-16
BICDL2	6.065168582	9.72E-22
TAF11L2	6.070753436	4.59E-22
ENSG00000289525	6.072061646	1.46E-26
ARL8BP2	6.113356906	4.62E-20
ENSG00000234292	6.131618465	1.47E-48
ENSG00000259962	6.189809169	3.85E-22
CSF2RBP1	6.280241907	1.08E-36
TMEM252	6.304164328	7.12E-22
ENSG00000289927	6.466661631	3.06E-37
ALOX15	6.492130366	8.40E-44
ENSG00000255097	6.494979441	7.14E-22
ENSG00000289251	6.520456526	4.19E-101
ENSG00000287586	6.528708459	2.33E-38
ENSG00000287771	6.647589617	4.93E-42

ENSG00000288875	7.549652238	5.69E-82
ENSG00000289538	7.615107932	2.08E-36
ENSG00000289636	7.764844623	9.33E-29
SIGLEC8	8.581783793	1.51E-42
ENSG00000275371	8.649582747	1.07E-53
