# Supplementary material for: Transcriptome Study in Sicilian Patients with Huntington’s Disease
Source: Diagnostics (Basel). 2025 Feb 7;15(4):409. doi: 10.3390/diagnostics15040409 (PMC11854416; doi:10.3390/diagnostics15040409)
Supplement: Supplementary file 1 [file diagnostics-15-00409-s001.zip › Supplementary_Table S2.pdf]

Supplementary Table S2. The table highlights the analysis ratio of the “Enrichment in Phenotype” section for the results of gene sets with a positive enrichment score.

**NAME**	**NAME_GO**	**SIZE**	**ES**	**NES**	**NOM p-val**	**FDR q-val**	**FWER p-val**
OXIDOREDUCTASE_ACTIVITY	GOMF_OXIDOREDUCTASE_ACTIVITY	40	0.39502034	1.8050942	8.99E-03	1.00E+00	1.00E+00
RNAI_EFFECTOR_COMPLEX	GOCC_RNAI_EFFECTOR_COMPLEX	18	0.49956915	1.8133866	1.11E-02	1.00E+00	1.00E+00
ABNORMAL_STERNUM_MORPHOLOGY	HP_ABNORMAL_STERNUM_MORPHOLOGY	16	0.4823862	1.7015623	2.26E-02	1.00E+00	1.00E+00
NUCLEOSOME	GOCC_NUCLEOSOME	15	0.4702415	1.6254281	2.63E-02	1.00E+00	1.00E+00
REGULATORY_NCRNA_MEDIATED _GENE_ SILENCING	GOBP_REGULATORY_NCRNA_MEDIATED _GENE _SILENCING	26	0.4066802	1.6408	2.69E-02	1.00E+00	1.00E+00

Legend: SIZE, number of genes in the gene set after filtering out those genes not in the expression dataset. **ES**, Enrichment score for the gene set; that is, the degree to which this gene set is overrepresented at the top or bottom of the ranked list of genes in the expression dataset. **NES**, Normalized enrichment score; that is, the enrichment score for the gene set after it has been normalized across analyzed gene sets. **NOM p-val**, Nominal p value; that is, the statistical significance of the enrichment score. The nominal p value is not adjusted for gene set size or multiple hypothesis testing; therefore, it is of limited use in comparing gene sets. **FDR q-val**, False discovery rate; that is, the estimated probability that the normalized enrichment score represents a false positive finding. **FWER p-val**, Familywise-error rate; that is, a more conservatively estimated probability that the normalized enrichment score represents a false positive finding. Because the goal of GSEA is to generate hypotheses, the GSEA team recommends focusing on the FDR statistic.
